# Investigating active phase loss from supported ruthenium catalysts during supercritical water gasification†

**DOI:** 10.1039/d1cy00379h

**Published:** 2021-10-14

**Authors:** Christopher Hunston, David Baudouin, Mohamed Tarik, Oliver Kröcher, Frédéric Vogel

**Affiliations:** Bioenergy and Catalysis Laboratory, Paul Scherrer Institut (PSI) 5232 Villigen PSI Switzerland david.baudouin@psi.ch +41 563105694; Institute of Chemical Sciences and Engineering (ISIC), École Polytechnique Fédérale de Lausanne (EPFL) 1015 Lausanne Switzerland; Institute for Biomass and Resource Efficiency, Fachhochschule Nordwestschweiz (FHNW) 5210 Windisch Switzerland

## Abstract

Active phase loss mechanisms from Ru/AC catalysts were studied in continuous supercritical water gasification (SCWG) for the first time by analysing the Ru content in process water with low limit-of-detection time-resolved ICP-MS. Ru loss was investigated alongside the activity of commercial and in-house Ru-based catalysts, showing very low Ru loss rates compared to Ru/metal-oxides (0.2–1.2 *vs.* 10–24 μg g_Ru_^−1^ h^−1^, respectively). Furthermore, AC-supported Ru catalysts showed superior long-term SCWG activity to their oxide-based analogues. The impact on Ru loss of several parameters relevant for catalytic SCWG (temperature, feed concentration or feed rate) was also studied and was shown to have no effect on the Ru concentration in the process water, as it systematically stabilised to 0.01–0.2 μg_Ru_ L^−1^ for Ru/AC. Looking into the type of Ru loss in steady-state operation, time-resolved ICP-MS confirmed a high probability of finding Ru in the ionic form, suggesting that leaching is the main steady-state Ru loss mechanism. In non-steady-state operation, abrupt changes in the pressure and flow rate induced important Ru losses, which were assigned to catalyst fragments. This is directly linked to irreversible mechanical damage to the catalyst. Taking the different observations into consideration, the following Ru loss mechanisms are suggested: 1) constant Ru dissolution (leaching) until solubility equilibrium is reached; 2) minor nanoparticle uncoupling from the support (both at steady state); 3) support disintegration leading to the loss of larger amounts of Ru in the form of catalyst fragments (abrupt feed rate or pressure variations). The very low Ru concentrations detected in process water at steady state (0.01–0.2 μg_Ru_ L^−1^) are close to the thermodynamic equilibrium and indicated that leaching did not contribute to Ru/AC deactivation in SCWG.

## Abstract 2

Ru loss mechanisms were investigated for the first time in SCWG by ICP-MS. Ru leaching at steady state was very low, close to thermodynamic models. Abrupt changes in process conditions must be avoided to prevent catalyst damage and higher Ru loss.

## Graphical abstract

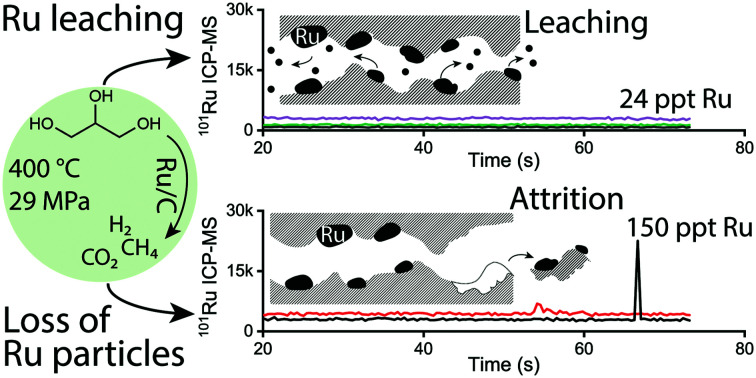

## Introduction

Catalytic hydrothermal gasification (cHTG) has been gaining more and more interest over the past decade as an alternative process for renewable gas production. This is due to the technology's ability to fully convert a variety of wet biomass streams into a methane- or hydrogen-rich gas.^1–6^ cHTG takes advantage of the physico-chemical properties of water in the near- and supercritical state. Radical changes in the density and dielectric constant of water cause supercritical water (SCW) to behave as an organic, non-polar solvent. As a result, SCW exhibits very low solubility for salts, but very high solubility for light gases and organic molecules, which along with the low viscosity of SCW decreases mass-transfer related issues for the catalytic conversion of organic molecules.^7,8^ Salts precipitate under SCW conditions and can therefore be extracted from the process, allowing the recovery of minerals (*e.g.* phosphates) and protection of the catalyst bed downstream.^9,10^ Thanks to the use of an active gasification catalyst, carbon-depleted water comes out as effluent. This process can thus transform waste biomass streams into renewable natural gas (bio-SNG), clean water and a brine effluent where most minerals have been extracted and concentrated. Although the process is run under harsh conditions (22–35 MPa, 374–500 °C), it becomes energetically more efficient to convert biomass streams with moisture contents above 70–80 wt% in SCW compared to conventional thermochemical conversion technologies, which would require energy-intensive drying steps.^11–13^

In this work, the focus is set on low-temperature SCW (374–500 °C) using an active catalyst in order to selectively form CH_4_, thermodynamically favoured at these temperatures (higher temperatures will favour H_2_ formation).^12,14–18^ In this temperature range, biomass readily hydrolyses in the absence of a catalyst. To ensure high carbon gasification efficiency and methane selectivity, a stable and active methanation catalyst is required.^12,19^ Many support materials have been investigated for their stability in SCW, but unfortunately only a very narrow selection remains stable (physically and structurally). The conclusions from those different studies are that only α-Al_2_O_3_, rutile-TiO_2_, monoclinic-ZrO_2_, CeO_2_, Ce–ZrO_2_ and activated carbon (AC) can be considered as stable materials for supercritical water gasification (SCWG).^12,18,20–24^ Regarding the active metal, ruthenium was shown to be very active in SCWG, while allowing high selectivity towards CH_4_ formation.^5,12,19,22,25^ Non-noble metal alternatives such as nickel are also active, however sintering, oxidation of the Ni phase as well as higher Ni solubility (Ni_30MPa,400°C_: 10^−5^ mol kg^−1^, Ru_30MPa,400°C_: 10^−12^ mol kg^−1^)^26^ compared to Ru are major issues under these conditions.^20,24,27–30^ In addition, metal oxide-supported Ru catalysts have been shown to be much less stable than carbon-based catalysts for continuous SCWG. The exact cause for this still remains unclear.^31^ Possible reasons could be the difference in the support specific surface area (SSA), or the different surface chemistry of the supports.^32^ Ru/AC was thus shown to be the most active and stable catalyst for long-term continuous SCWG.^22,31^ Mechanistic studies were performed to understand the role of Ru nanoparticles (Ru NPs) in SCWG and the nature of the active phase. Under these conditions, the active phase is readily reduced by organics (ethanol) at 150 °C as shown by X-ray absorption spectroscopy (XAS).^33^ However, the final reduced Ru fraction reached 95% at 400 °C, suggesting the presence of 5% of ruthenium oxide either in the bulk or at the surface of the particles. Given the extreme SCW conditions and the reactants involved, surface oxygen and hydroxide groups are expected to be present at the metal surface as evidenced by D_2_O splitting on Ru.^34^ The reduced Ru was also shown by XAS to remain stable with no reoxidation occurring in pure SCW at 350 °C.^35^ The activation of CH_4_ on reduced Ru NPs and the subsequent scrambling mechanism was proven very rapid in SCWG and outlines that the conditions remain highly reducing throughout the catalyst bed.^33^

**Fig. 1:**
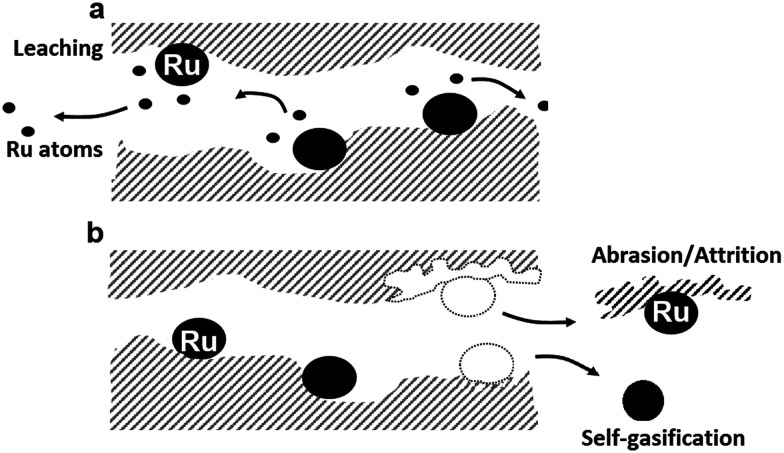
Schematic view of Ru loss mechanisms from the AC catalyst support. a) Ru leaching, b) loss of support fragments leading to the loss of Ru NPs, either from self-gasification of the support or from abrasion/attrition between catalyst grains (mechanical loss).

The stability of Ru, an expensive noble metal, is of crucial importance for economically viable commercialisation of the technology. Common liquid-^36,37^ and gas-phase^38,39^ deactivation mechanisms also occur in SCWG. For example, Arena^37^ showed that iron and sulphur poisoned a Ru/Al_2_O_3_ catalyst during continuous glucose hydrogenation. Iron was also shown to remain stable as porphyrin complexes during hydrothermal liquefaction (HTL) of microalgae,^40^ with Ru/AC showing the same efficiency as AC in removing Fe from hemin.^41^ In long-term continuous SCWG operation, the catalyst will eventually be prone to deactivation too, which can occur in different forms: poisoning (sulphur, transition metals), fouling (coke or salt deposition), sintering, change in the nature of the active phase, “self-gasification of the support, and mechanical or chemical loss of the active phase.^6,42–44^ Active Ru/AC catalysts tested in the kinetic regime with model feeds still show deactivation patterns.^31,45^ In these cases, poisoning and fouling other than from carbon can be left out of the equation. The remaining deactivation mechanisms using model solutions are thus fouling by coke deposition and sintering of the active phase (although limited with Ru),^27^ as well as loss of the active phase. The latter can occur through different mechanisms, namely through chemical or mechanical losses as schematised in Fig. 1. A chemical Ru loss refers to the dissolution of the active phase into the solvent (SCW), commonly known as leaching. Fortunately, Ru (metallic and oxide forms) is one of the least soluble metals active in SCW according to a thermodynamic dissolution modelling analysis in hydrothermal media.^24,26^ However, some Ru complexes (*e.g.* acetate, oxalate) are poorly soluble in water under ambient conditions, suggesting that they may exhibit increased solubility in SCW. Unfortunately, no solubility data are available for such complexes in SCW. However, these organo–Ru complexes are thought to decompose through hydrolysis and oxidation to Ru^(0)^ or RuO_2_ similarly to other organometallic complexes in SCW.^46–50^ Another form of chemical Ru loss can occur through “self-gasification” of the carbon support by Ru NPs.^43^ As Ru is a very active metal capable of breaking down organics by C–C bond cleavage, it can also catalyse the gasification of activated carbon to which it is anchored (*i.e.* self-gasification). This could lead to the loss of Ru NPs instead of ionic Ru. Similarly, weakly bound Ru clusters can also be flushed out of the system. Mechanical Ru losses can occur through the loss of support domains containing anchored Ru NPs, because of abrasion or attrition. Although most granular activated carbons remain mechanically stable, they are still brittle materials and SCW conditions can lead to abrasion/attrition of the support,^43^ inducing the loss of the active phase from the catalyst bed.

Recently, appreciable amounts of Ru (mg_Ru_ L^−1^, or ppm range) were detected in process water after cHTG of an aqueous effluent from hydrothermal liquefaction (HTL) over a Ru/AC bed.^51^ Major concerns arose from these figures regarding the economic viability of cHTG. It is important to keep in mind that a gasification catalyst must maintain its activity over at least 8000 h per year for commercial SCWG operation.^52^ These results led us to investigate our catalytic systems more thoroughly by accurately monitoring the Ru loss during SCWG in order to understand the mechanisms involved and find solutions to circumvent this Ru loss.

## Experimental

### Materials and methods

In-house supported Ru catalysts were prepared on activated carbon (Jacobi Carbons), α-Al_2_O_3_ (Alfa Aesar), rutile-TiO_2_ (Norpro Saint-Gobain) and monoclinic-ZrO_2_ (Norpro Saint-Gobain). The catalysts were synthesised by wet impregnation (4 h, 40 °C, continuous agitation) of the sieved supports (0.5–0.8 mm) with an aqueous 1.5% (wt/vol) RuNO(NO_3_)_3_ solution (Alfa Aesar). The suspension was then evaporated at 60 °C by progressively decreasing the pressure on a rotary evaporator. The catalysts were left at RT and 150 mbar overnight to fully evaporate the solvent. The impregnated supports were then dried overnight in an oven at 110 °C in air. The metal oxide-supported catalysts were calcined in a quartz tube at 450 °C (10 °C min^−1^, 4 h) in N_2_/O_2_ (80 : 20, 250 mL min^−1^). Eventually, all catalysts were reduced at 400 °C (5 °C min^−1^, 6 h) in a flow of N_2_/H_2_ (80 : 20, 500 mL min^−1^). After reduction, they were passivated by letting air diffuse through the open reactor. The catalysts obtained were used without further treatment. Three commercial catalysts – 5 wt% Ru supported on activated carbon, Ru/AC-BM (BASF), Ru/AC-A, and Ru/AC-B (both proprietary) – were also evaluated in this study. Ru/AC-BM is the benchmark catalyst used for gasification campaigns on PSI's cHTG demonstration unit.^6,53^

### Continuous SCWG setups

The catalytic testing was performed on a SCWG setup referred to as Konti-I (modified from previous studies).^23,27^ The feed, aqueous glycerol (2.5–20 wt%) (diluted from an 85 wt% solution (Kuhn AG)), was pumped into the system with a high-pressure pump (Knauer 80P) and heated to reach 400 °C at the entry of the catalyst bed. A series of three heaters was used to heat the feed to the required reaction temperature (pre-heater and transfer heater upstream of the catalytic reactor, followed by a tube furnace around the tubular reactor (Carbolite)). The fixed-bed plug-flow reactor (*L* = 460 mm, i.d. = 8 mm, o.d. = 14.3 mm), made of 316L stainless steel (SITEC-Sieber Engineering AG), was filled with a catalyst (0.97–1.07 g, 24–49 mm bed height, depending on the bulk density). α-Al_2_O_3_ beads (0.8 mm diameter, 0.03 cm^3^ g^−1^ porosity, Alfa Aesar) were loaded upstream of the catalyst bed (4.33–6.01 g, 130–175 mm bed height), acting as an inert filling material. The feed entered the reactor and flowed downwards first through the inert filling material and then through the catalyst bed before exiting the reactor. Three sizes of stainless steel wire mesh – 0.08, 0.16 and 0.25 mm – held the packed bed at the desired height in the tubular reactor. The reactor effluent was cooled down with a heat exchanger and forced through a 15 μm frit to protect the valves downstream. A back pressure regulator (Tescom) maintained the system at the desired pressure (29 MPa). The reactor effluent eventually entered a phase separator, from where the water and gas exited the setup. The latter flowed through a Peltier cooler (1–4 °C) to condense the water out of the gas before being analysed online with a μGC (Inficon). An automated sampler was used to collect the liquid effluent (10–15 mL) at defined times on stream (5 min sampling time). Samples were taken every hour during daytime operation and every 2.5 h for night operation to monitor the carbon and ruthenium concentrations. An additional sample was taken before the start of each experiment during the reactor-flushing phase (deionised (DI) water, ambient conditions). A blank SCWG experiment (400 °C, 29 MPa, 5 g min^−1^) was performed by feeding 10 wt% glycerol over activated carbon (Ru/AC-BASF catalyst support) for Ru quantification in the effluent.

The gasification experiments were divided into different sections, according to the WHSV_g_Ru__ or temperature at which they were run. The weight-hourly space velocity normalised per gram Ru (WHSV_g_Ru__) was used as a space velocity unit to facilitate the comparison between different catalysts and experiments. The catalyst testing systematically began at the lowest WHSV_g_Ru__ (500–600 g_Org_ g_Ru_^−1^ h^−1^), at which benchmark catalysts achieve full carbon conversion and produce gases matching the thermodynamic equilibrium composition.^53^ Once a steady-state section was reached for a few hours, the catalyst was evaluated under different process conditions (*i.e.* WHSV_g_Ru__, temperature, concentration). The WHSV_g_Ru__ was varied by either changing the feed rate (5–20 mL min^−1^) or the feed concentration (2.5–20 wt%). For night operation, the WHSV_g_Ru__ was systematically set back to its lowest value (≈600 g_Org_ g_Ru_^−1^ h^−1^).

Two other setups were used for the SCWG of undigested sewage sludge (total solids = 2.6%, total organic carbon (TOC) = 11.7 g L^−1^, total inorganic carbon (TIC) = 0.66 g L^−1^, pH = 6.9). Both were equipped with a salt separator before the catalytic reactor to extract most of the minerals from the sewage sludge.^54^ A commercial sulphur absorbing material (Johnson Matthey) was also used upstream of the catalyst bed to prevent catalyst poisoning.^55^ One setup is referred to as an intermediate setup (30 g S-absorber, 50 g catalyst) and the other one as Konti-C (280 g S-absorber, 550 g catalyst). The latter is PSI's 1 kg h^−1^ catalytic hydrothermal gasification demonstration unit, which is used to perform biomass gasification campaigns.^53^ The setup characteristics are summarised and compared in Table 1.

**Table 1:** Characteristics of the different SCWG setups and main experimental conditions used

SCWG setup	Reactor dimension i.d./*L*^a^ (mm)	Feed rate (L h^−1^)	Catalyst mass (g)	Catalyst grain size (mm)	WHSV_g_Ru__		*T* (°C)	*P*_avg_ (MPa)	*X*_C_ b (%)
					Glycerol	Sludge			
					(g_Org_ g_Ru_^−1^ h^−1^)				
Konti-I	8/460	0.3^c^	1	0.5–0.8	600^c^	—	400	29	99.9
Intermediate	12/1435	1	50	0.8–1.25	16	5	400	25	99.9
Konti-C	36/1515	1	550	2.0–4.0	—	0.4	400^d^	26	99.9

a i.d. = inner diameter, *L* = length.

b Carbon conversion over the run duration.

c Value at the start of an experiment, before variation (lowest feed rate and WHSV_g_Ru__).

d Average between the inlet and outlet temperatures, *T*_in_ = 390 °C, *T*_out_ = 410 °C.

### Analytics

The BET specific surface area (SSA) and the total pore volume were measured by N_2_ physisorption (77 K) on an Autosorb-1 (Quantachrome). The samples were outgassed in dynamic vacuum for a minimum of 4 h at 300 °C. SSA_Tot_ was calculated according to the BET model, the total pore volume was determined at relative pressures *p*·*p*_0_^−1^ ≥ 0.99. The micropore volume (*V*_MP_) was determined by the t-method developed by Lippens and de Boer.^56^ The non-micropore volume is defined here as the external volume (*V*_Ext_) and is calculated by subtracting the micropore volume from the total pore volume: *V*_Ext_ = *V*_Tot_ − *V*_MP_. The same calculation applies for the external surface area (SSA_Ext_) that defines the sample surface area without the micropore contribution. The ruthenium loading of the in-house catalysts was determined by mass balance after the impregnation procedure (eqn (1))1
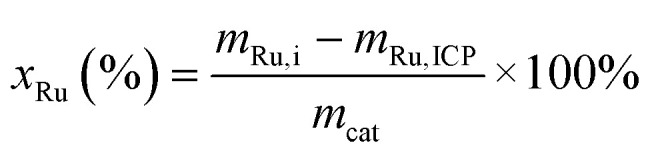
where *x*_Ru_ is the final loading, *m*_Ru,i_ is the mass of elemental Ru used for the impregnation, *m*_Ru,ICP_ is the Ru loss determined by ICP-MS and *m*_cat_ is the final mass of the catalyst. 20 mL of MilliQ water (18.2 MΩ cm) were used to wash the impregnation flasks to dissolve the remaining Ru precursor salts, the solution was then analysed by inductively coupled plasma-mass spectrometry (ICP-MS). The Ru dispersion (*D*) was measured by CO pulse titration on a TPD/R/O 1100 (Thermo Scientific). First, temperature-programmed reduction (TPR) was performed in 5% H_2_/Ar (300 °C, 5 °C min^−1^, 1 h hold), followed by treatment in He 6.0 (400 °C, 1 h) to remove the remaining H_2_, eventually the catalyst was cooled down to 25 °C and titrated with pulses of 20% CO/He. A CO : Ru stoichiometric factor of 1 was assumed for the dispersion calculations, and confirmed by static H_2_ chemisorption measurement yielding a dispersion within the error of the CO method. The average Ru particle size (*d*_p_) was calculated from the dispersion with the equations developed by Borodziński and Bonarowska.^57^ A detailed description can be found in the work of Peng *et al.*^31^

The gas produced from SCWG was analysed online with a μGC 3000 series (Inficon) having two different columns with TCDs. The first column (Molsieve, 10 m × 320 μm × 30 μm) analyses H_2_, O_2_, N_2_, CH_4_ and CO in He as carrier gas at 120 °C, 25 psi. The second column (PLOTQ, 8 m × 320 μm × 10 μm) analyses CO_2_, H_2_S, C_2,3_ in Ar as carrier gas at 70 °C, 20 psi. The carbon gasification efficiency GE_C_ can be determined by eqn (2)2
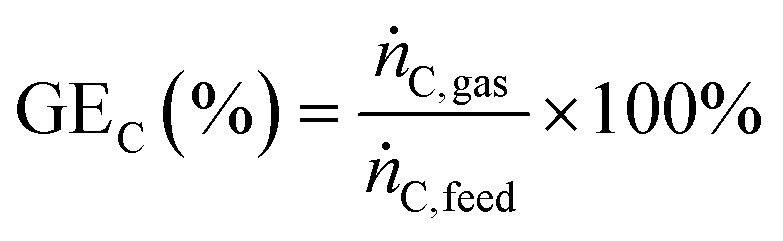
by knowing the total amount of carbon converted to gas (*ṅ*_C,gas_) and the flow of carbon entering the system (*ṅ*_C,feed_) per unit of time.

The collected liquid effluent was analysed for its carbon content on a Dimatoc2000 (DIMATEC). The instrument measures the total carbon (TC) by oxidising all the carbon into CO_2_ at 850 °C in a quartz reactor containing a 5% Pt/SiO_2_ catalyst. The total inorganic carbon (TIC) is determined by converting the carbonates to CO_2_ at 160 °C by adding phosphoric acid in a quartz reactor containing porous silica gel beads. The total organic carbon (TOC) is eventually determined by subtraction (TOC = TC − TIC). The carbon conversion (*X*_C_) is calculated from eqn (3):3
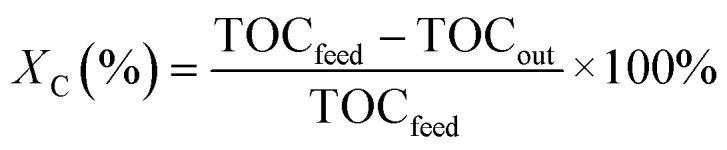
where TOC_feed_ and TOC_out_ are the organic carbon contents of the feed and process water, respectively.

X-ray diffraction spectra were measured on a D8 Advance (Bruker) diffractometer with Cu Kα_1_ radiation (*λ* = 1.5406 Å) and a 1D-LynxEye detector.

The apparent turnover frequency (TOF_app_) (eqn (4)) was used to compare the activity of a catalyst. The term “apparent” is used because the dispersion decreases during the SCWG experiments, and also because it is calculated at carbon conversions between 50 and 80%, which are above the ones usually used in kinetic studies.4
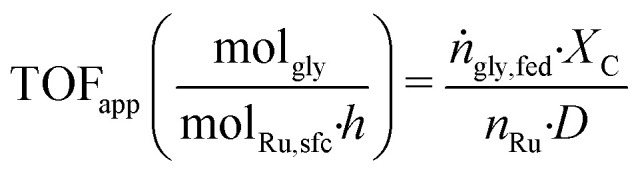
where *ṅ*_gly,fed_ is the mole flow rate of glycerol, *n*_Ru_ is the moles of Ru in the catalyst and *D* is the Ru dispersion.

ICP-MS was used to determine the Ru, Al, Ti, Zr concentrations in the SCWG process water. All original samples (non-filtered) were acidified by adding high purity HNO_3_ (Ultratrace grade, Fischer Scientific) to reach a final HNO_3_ concentration of 1%. Commercial single-element standard solutions of Ru, Al, Ti, Zr and a 1% HNO_3_ dilution solution were used for the preparation of the calibration standards with concentrations of 0, 5, and 50 μg L^−1^ (ppb) for Al, Ti and Zr elements, and concentrations of 0, 20, 200 and 2000 ng L^−1^ (ppt) for Ru. For samples having Ru concentrations above the Ru calibration range (10–2000 ng L^−1^), dilution steps were performed using a 1% HNO_3_ solution. Milli-Q water was used for all sample and standard solutions. The analysis was carried out on an ICP-MS 7700x (Agilent). Prior to each analysis, daily optimisation of the ICP parameters was performed by using a multi-element standard containing Li, Mg, Co, Y, Tl and Ce to reach a high sensitivity and low oxidation rate (CeO^+^/Ce^+^ < 1%). The ^99^Ru and ^101^Ru isotopes were measured for all standard and sample solutions, either in the time-resolved mode (transient signal, an average was calculated over 50 s for each standard and sample solution) or in the normal mode (integration time of 0.2 s). The ^27^Al, ^47^Ti, pt49Ti, ^90^Zr and ^91^Zr isotopes were measured in the normal mode. A summary of the parameters as well as an example of some figures of merit (obtained in one of the Ru analyses) are listed in Table S1 of the ESI.† The relative standard deviation (RSD) of the average concentration values of all standard samples was below 5%.

## Results and discussion

### Continuous SCWG with commercial 5 wt% Ru/AC catalysts

**Fig. 2:**
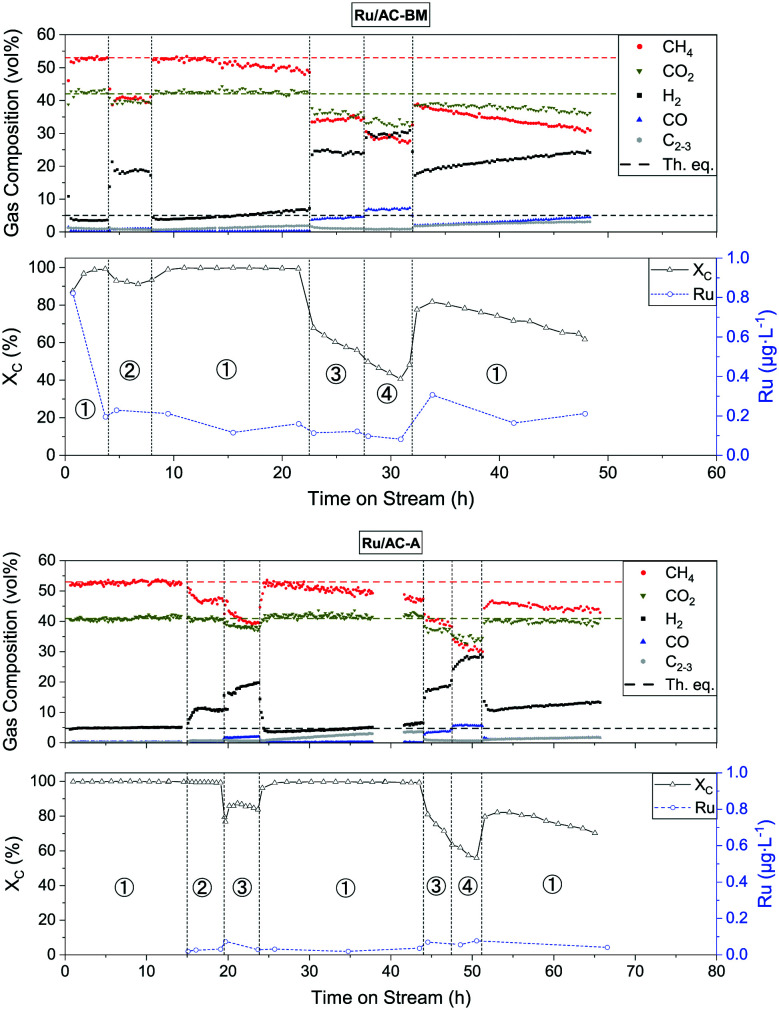
Catalytic testing of Ru/AC-BM (top) and Ru/AC-A (bottom). The produced gas (top part of the graphs) and both the carbon conversion and Ru concentration (bottom part of the graphs) are shown as a function of the time on stream (glycerol fed from TOS = 0 h onwards). Horizontal dashed lines indicate the thermodynamic equilibrium gas composition. Conditions: *T* = 400 °C, *p* = 29 MPa, WHSV_g_Ru__ ≈ 500–600, 1200, 1800, and 2400 g_Org_ g_Ru_^−1^ h^−1^ for sections 1–4, respectively.

The catalytic performance of different commercial catalysts was assessed by gasifying an aqueous solution of 10 wt% glycerol. The characteristics of the tested catalysts are summarised in Table 2. First, the SCWG performance of the three commercial catalysts was compared at different space velocities. An overview of the experiments with Ru/AC-BM and Ru/AC-A is shown in Fig. 2 (for Ru/AC-B, see Fig. S1†). The top part of the graphs highlights the gas composition with regard to time on stream (TOS). The bottom part indicates both the carbon conversion (*X*_C_) and the measured ruthenium concentration in the effluent stream. Changes in WHSV_g_Ru__ by modifying the feed rate are depicted by the dashed vertical lines. The circled numbers relate to sections at constant WHSV_g_Ru__ (1: 500–600, 2: 1200, 3: 1800, 4: 2400 g_Org_ g_Ru_^−1^ h^−1^). Previous experiments on both catalysts showed similar trends, with the CH_4_ selectivity starting to drop in section 2 and the CO selectivity increasing as soon as *X*_C_ drops. The latter started decreasing from sections 2 and 3 onwards, indicating a generally good repeatability of the catalytic experiments.

**Table 2:** Main characteristics of the fresh 5 wt% Ru/AC commercial catalysts

Catalyst	*x*_Ru_ a (%)	*D* b (%)	*d*_p_ c (nm)	SSA_Tot_ (m^2^ g^−1^)	SSA_MP_ (m^2^ g^−1^)	SSA_Ext_ (m^2^ g^−1^)
5% Ru/AC-BM	5.0	32 ± 4	3.6 ± 0.6	1172 ± 8	1107 ± 13	66 ± 15
5% Ru/AC-A	5.3	40 ± 5	2.7 ± 0.5	1145 ± 26	1114 ± 26	31 ± 37
5% Ru/AC-B	5.0	13	9.7	1258	1202	56

a Metal loadings from the suppliers' product specification.

b Ru dispersion measured by CO pulse titration.

c Calculated from *D*.^57^

In section 1 of the testing phase (WHSV_g_Ru__ ≈ 500–600 g_Org_ g_Ru_^−1^ h^−1^), both catalysts achieved carbon conversions above 99.9% and the gas composition reached the thermodynamic equilibrium (53% CH_4_, 4% CO_2_, 6% H_2_, 0.05% CO).^23^ When doubling the space velocity (section 2), the conversion decreased to 90% for Ru/AC-BM and the gas composition changed too; CH_4_ selectivity decreased in favour of H_2_. For Ru/AC-A, full carbon conversion was achieved in sections 1 and 2 (WHSV_g_Ru__ ≈ 500–600 and 1200 g_Org_ g_Ru_^−1^ h^−1^). Though the conversion was still above 99.9% in section 2, the methane concentration dropped while hydrogen increased and CO_2_ remained unaffected. Section 3 shows the first drop in conversion for Ru/AC-A, the methane fraction decreased in favour of H_2_ again. After sections 2 and 3, the catalyst was brought back to the conditions of section 1, where it managed to convert >99.9% of the feed, as does Ru/AC-BM. For both catalysts, the gas compositions are shifted back to equilibrium, but started to drift away after a few hours. This loss in methane selectivity at the set WHSV_g_Ru__ is the first evidence of catalyst deactivation. When pushing the catalyst to higher WHSV_g_Ru__ (sections 3 and 4), the conversion further decreased to approximately 40% for Ru/AC-BM and 50% for Ru/AC-A. At these conversions, the deactivation trend can clearly be observed. The gas compositions continued to be affected, *i.e.* the CH_4_ selectivity decreased, replaced mainly by H_2_ (20–30%) and CO (5–10%). Interestingly, higher CO concentrations appeared as soon as the feed was not fully converted, as observed in sections 3 and 4. This coincided with the clear deactivation trends observed for both catalysts. Eventually both runs ended with section 1, in which the carbon conversion indicated the same deactivation trend for both catalysts. Ru/AC-A and Ru/AC-BM exhibited similar SCWG activity, with the former performing slightly better (evidenced in sections 2 and 3), maybe due to the small difference in loading (see Table 2). For the type of model feed used in this work, the upper WHSV_g_Ru__ limit at which the system remained in the thermodynamic regime (*i.e.* >99.9% conversion and equilibrium gas composition) lies between 600 and 1200 g_Org_ g_Ru_^−1^ h^−1^.

The catalysts were characterised before (fresh) and after the catalytic testing (spent), and their SSA is reported in Table 3. The total surface area (SSA_Tot_) was drastically affected, with losses of 50% for Ru/AC-BM and Ru/AC-B. The data show that the decrease in SSA_Tot_ was mainly due to the loss of micropores. This was likely due to carbon deposition in the pore network of the support, inducing filling of the micropores. Interestingly, the external surface area (SSA_Ext_) increased for all spent catalysts. Partial collapse of the pore structure, partial gasification of the support or coke deposition could be the reasons for this increase. Other analysis techniques such as temperature-programmed oxidation or infrared spectroscopy are commonly used to assess carbon deposits on catalysts, however this is not helpful with carbon supports.

**Table 3:** SSA comparison between the fresh and spent catalysts after SCWG of glycerol

Catalyst	SSA_Tot_	SSA_MP_	SSA_Ext_	SSA_Tot_	SSA_MP_	SSA_Ext_	ΔSSA_Tot_^a^ (%)
	Fresh (m^2^ g^−1^)			Spent (m^2^ g^−1^)			
5% Ru/AC-BM	1172 ± 8	1107 ± 13	66 ± 15	605	527	78	−50
5% Ru/AC-A	1145 ± 26	1114 ± 26	31 ± 37	1086	950	136	−5
5% Ru/AC-B	1258	1202	56	621	467	157	−51

a SSA_Tot_ loss reported as a percentage based on the fresh material.

Liquid samples (5 min sampling time) were analysed by ICP-MS to monitor the Ru concentration in the liquid effluent and track the Ru loss from the catalytic bed. The measured Ru concentration is reported as a function of time on stream in Fig. 2 (bottom graphs). For Ru/AC-BM, the first collected sample contained 0.8 μg L^−1^ Ru, but quickly decreased to 0.2 μg L^−1^ Ru. This high initial concentration was probably due to the nature of the catalyst support. Although very stable in SCW, AC remains a brittle material, hence the loss of unstable carbon domains or loss of the least strongly anchored Ru NPs could explain the higher initial Ru loss. After this initial decrease, the Ru concentration for Ru/AC-BM stabilised around 0.20 ± 0.06 μg L^−1^ Ru, whereas for Ru/AC-A it was significantly lower at 0.03 ± 0.01 μg L^−1^ Ru (Ru/AC-B: 0.08 ± 0.05 μg L^−1^ Ru). Blank concentrations amounted to 11.5 ± 6.5 ng L^−1^ (LOD_101Ru_ = 1.08 ng L^−1^, see Table S1†), obtained by analysing the process water after SCWG of a 10 wt% glycerol solution over the AC support only. Similar metal loss trends were observed for Ni/AC and Ni/γ-Al_2_O_3_ by Wang *et al.*^58^ when gasifying phenol in the presence of O_2_ (480 °C, 25 MPa). Ni concentrations were in the range of 10–15 mg L^−1^ after 1 h TOS, before stabilising around 1 mg L^−1^ after 30 h. Li *et al.*^59^ observed the same trend for Ni/α-Al_2_O_3_ when gasifying glycerol (425 °C, 25 MPa), with initial and final (24 h TOS) Ni losses of 280 and 10 mg L^−1^, respectively. These figures are at least 10^4^ times higher than that for Ru, highlighting the better stability of the latter metal.

The monitored Ru concentration in Fig. 2 was stable and remained similar throughout the sections, independent of conversion (bar the few spikes observed at TOS = 1 h and 33 h for Ru/AC-BM – see discussion below). Leaving the concentration spikes aside, the Ru signal remained stable and can be considered at steady state. This highlights that the WHSV_g_Ru__ did not affect the Ru concentration in the effluent stream. Although the ICP-MS data for the first samples of Ru/AC-A are not available, another Ru/AC-A catalyst sample was washed in DI water (3 h with continuous agitation under ambient conditions) and filtered (7–12 μm filter paper) to mimic this initial washing phase. The resulting Ru concentration in water amounted to 6.5 μg L^−1^ Ru after the first wash and 1.5 μg L^−1^ Ru after the second one. This confirms that the initial sharp decrease in Ru concentration is the same as for Ru/AC-BM and that the same should be expected for other Ru/AC catalysts. The ICP-MS data show very low absolute Ru concentrations in the effluent streams. Converted into a normalised Ru loss rate, the figures amount to 1.2 ± 0.4 and 0.2 ± 0.1 μg_Ru_ g_Ru,bed_^−1^ h^−1^ for Ru/AC-BM and Ru/AC-A, respectively (Ru/AC-B: 0.4 ± 0.3 μg_Ru_ g_Ru,bed_^−1^ h^−1^ – see Fig. S1†). The values were calculated for section 1, since it is most representative of industrial operation (lowest WHSV_g_Ru__, thermodynamic regime). The Ru quantified in the effluent stream did not seem to be directly involved in catalyst deactivation because of the negligible amount lost with regard to the total surface (sfc) Ru present in the catalyst bed (0.47 ± 0.15 μmol_Ru,lost_ mol_Ru,sfc,bed_^−1^ h^−1^). The loss in TOF_app_ observed during the second step 3 (TOS = 44–47 h) was 5 mol_gly,converted_ mol_Ru,sfc,bed_^−1^ h^−1^ (amounting to a 12% decrease in TOF_app_ during section 3). The exact same decrease was observed for section 4. Hence, the Ru loss had a negligible effect on the loss in conversion observed at high WHSV_g_Ru__ in sections 3 and 4. It is important to keep in mind that the methanation reaction over Ru is size-sensitive.^60,61^ Hence, the loss of small NPs (being the most active because of the increased presence of *B*_5_ sites)^62^ would lead to a rapid loss in methanation activity, which was not observed here, suggesting that leaching would be the main Ru loss mechanism. The exact cause of catalyst deactivation remains unknown, but the loss in surface area of up to 50% for the catalysts tested with glycerol on Konti-I is in line with coking. Recent investigations showed that carbon deposits on Ru/AC catalysts used for crude glycerol conversion could be extracted, which led to an increase in SSA as well as a small regain of activity.^44^ Hence, coking is thought to be one of the deactivation mechanisms under model feed conditions. The behaviour of the time-resolved ICP-MS signal may help in understanding the type of Ru loss the catalyst is facing, with different physico-chemical mechanisms occurring in parallel (*e.g.* leaching, mechanical loss of support). Typically, the presence of sudden spikes in the time-resolved signal could indicate the presence of Ru-rich particles (catalyst fragments, Ru clusters or NPs) in the liquid effluent (the samples were not filtered at any stage of the analysis). Conversely, a stable signal would be indicative of ionic Ru species in solution, given the measured concentration is above the level of quantification (LoQ). Therefore, time-resolved ICP-MS analyses were performed on selected process water samples from the three commercial catalyst tests. The signals for Ru/AC-A (Fig. S2†), Ru/AC-BM (Fig. S3†) and Ru/AC-B (Fig. S4†) were all relatively stable. Although fluctuations were observed, the RSD generally remained low (6–16% for Ru/AC-A, 3–8% for Ru/AC-BM, 5–17% for Ru/AC-B), indicating a low probability of finding large Ru NPs in the analysed samples. The higher RSDs mainly arose from the very low Ru concentrations (Fig. S5†). As a comparison, the Ru standard (0.5 μg L^−1^ Ru) closest to the measured concentrations had an RSD of 4.7% (Fig. S6†). This suggests that Ru was mainly present in the form of ions or small NPs. However, abrupt increases in Ru intensity were detected for Ru/AC-BM (Fig. S3/BM-5†) and Ru/AC-B (Fig. S4/B-2†), representing samples from TOS = 4.7 h and TOS = 1.6 h, respectively. The RSDs of both samples were higher than those of other samples in the same concentration range and can be seen as outliers in an RSD-concentration plot (Fig. S5†). This most probably indicated the presence of larger Ru particles or catalyst fragments in these liquid samples. If both outliers were used to estimate the fraction of larger particles released compared to the steady-state concentration (assumed as leaching), the part released from large particles would amount to 65% for Ru/AC-B-1.6 and 58% for Ru/AC-BM-4.7. Unfortunately, no information on the size of the analysed Ru particles (as in Lee *et al.*)^63^ can be extracted since measurements must be done in the single-particle mode. Due to the very porous nature of the catalyst support, it is not straightforward to conclude on the Ru loss mechanisms. Understanding the conditions favouring a rapid Ru loss is pivotal for commercial utilisation of cHTG. This triggered the need for a further in-depth study on the factors affecting Ru loss. Hence, a parametric study was performed to investigate and better understand the different mechanisms involved.

### Effect of process parameters on Ru loss

**Fig. 3:**
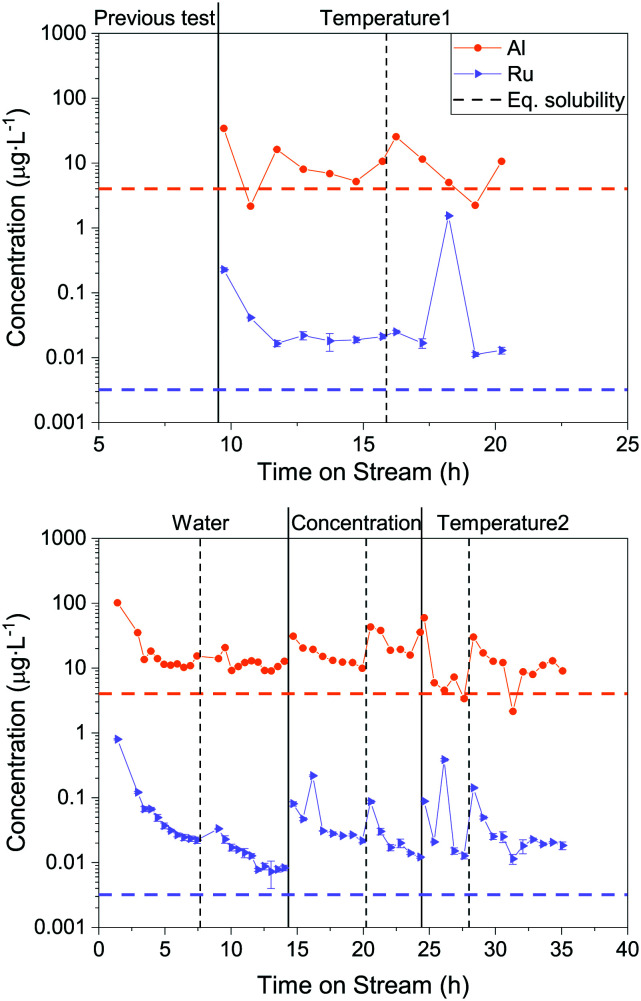
Overview of the parametric Ru loss study. Al and Ru concentrations were monitored over time on stream. 1st Ru/AC-A catalyst load (top) and 2nd Ru/AC-A catalyst load (bottom). Vertical lines indicate complete restarts of the setup (dashed line: same process parameter varied, full line: change in varied parameter). Horizontal dashed lines represent the thermodynamic equilibrium solubility of Al and Ru (400 °C, 30 MPa),^26^ respectively. The varied process parameter is indicated at the top of the graph. The pressure (29 MPa) and the WHSV_g_Ru__ (except for water) were kept constant (540–570 g_Org_ g_Ru_^−1^ h^−1^).

To understand how the Ru loss from the catalyst bed is affected by process parameters, the temperature, feed concentration and flow rate were systematically varied. In this process, it is not simple to vary only one parameter at a time, therefore the WHSV_g_Ru__ was maintained constant so that the organic molecules were exposed to the same number of Ru atoms per unit time. This implied adjusting the flow rate whenever the concentration was varied. This time, Ru and Al were both monitored in the process water over the duration of the experiments. The latter was also measured because of the use of α-Al_2_O_3_ beads as an inert filling material in the reactor upstream of the catalytic bed. This allowed investigating the stability of α-Al_2_O_3_ in SCW and seeing whether it could have a negative impact on the Ru/AC catalyst bed. All the results presented below come from gasifying aqueous glycerol solutions in the thermodynamic regime. Fig. 3 shows a generalised overview of the different tests. The results are split into two separate graphs because two different catalyst loads were used during this catalytic test (both Ru/AC-A). Looking at the Al concentration in the process water over the whole parametric study (Fig. 3), the concentration stabilised around 10 μg L^−1^, which is close to the modelled equilibrium solubility for aluminium oxide (4 μg L^−1^) according to Jocz and co-workers^26^ (Table S2†). It is important to note that the modelling equations were based on a worst-case scenario, meaning maximum irreversible catalyst dissolution into pure SCW. Data points with higher concentrations often occurred after restarts of the setup. Afterwards, the Al concentration progressively decreased towards this solubility equilibrium.

As for Al, the Ru concentration was highest right after changes in process parameters, but for Ru the variations were more pronounced. The Ru concentration then rapidly decreased – similarly to an exponential decay – before reaching a steady state. Unlike Al, the Ru concentration did not stabilise at the modelled solubility equilibrium (0.003 μg L^−1^), but between six and nine times above this limit. A few spikes in Ru concentration were also observed during these experiments. Note that these spikes can lead to a 100-fold increase in Ru concentration in the process water. Interestingly, the Ru concentrations seemed to stabilise towards similar numbers systematically, in the range 0.01–0.03 μg L^−1^. This holds true for both loads of catalyst tested (Ru/AC-A), indicating a good repeatability for Ru and Al quantification from the process water.

**Fig. 4:**
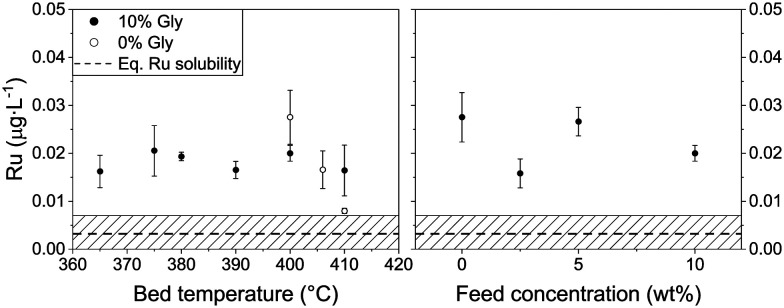
Mean Ru concentration in the process water for different reaction temperatures (left) and glycerol concentrations (right). Left: 10 wt% glycerol (full circles) and SCW (empty circles), feed mass flow rate = 5 g min^−1^. Right: Temperature = 400 °C, feed mass flow rate = 5, 20, 10, 5 g min^−1^ for feed concentrations of 0, 2.5, 5, 10 wt% glycerol, respectively. The residence time changed too, but it did not affect the Ru loss (see Fig. S8†). Concentration spikes were not accounted for in the mean calculations. Ru level of quantification (LoQ) = 0.007 μg L^−1^. Concentrations below the LoQ are represented by the hatched area. The pressure (29 MPa) and the WHSV_g_Ru__ (except for 0% glycerol) were kept constant (540–570 g_Org_ g_Ru_^−1^ h^−1^). The temperatures were averaged over the length of the catalyst bed. The horizontal dashed line represents the thermodynamic equilibrium solubility of Ru (400 °C, 30 MPa).^26^

Fig. 4 shows the mean Ru concentrations for the different steady states. It is important to note that the data points located in the initial steep concentration decrease, as well as sudden spikes in concentration were omitted for the statistical evaluation of the steady-state concentrations. The results presented in Fig. 4 show no specific trends between the Ru concentration and temperature or feed concentration. As a first guess, one would expect the Ru concentration to decrease after the critical temperature, since the SCW density and subsequently its static dielectric constant drastically decrease between 375 and 415 °C at 29 MPa.^64^ The dissolution model from Jocz *et al.*^26^ predicts a stable Ru concentration over the assessed temperature range (365–410 °C) in pure SCW. This is due to the increase in the equilibrium concentration of RuO, which compensates the concentration decrease of the ionic Ru species. Although the Ru concentration remained stable around 0.02 μg L^−1^, it remained six to seven times higher than the modelled thermodynamic dissolution equilibrium (0.003 μg L^−1^). This might be explained by the fact that the model described above was made for pure SCW and does not take into account the reducing environment of the SCWG experiments (aqueous glycerol solution converted into CH_4_, CO_2_ and H_2_). The produced gas can have an impact on the chemistry of ruthenium and certainly on the SCW-gas mixture density.^65^ These two parameters could have an effect on the dissolution equilibrium, but the measured Ru concentrations in experiments using different feed concentrations down to 0% glycerol rules out the influence of fluid composition or density. The comparison between the modelled^26^ and experimental data from this work indicated Ru loss from leaching, together with a loss of Ru particles.

To summarise, this parametric study showed that the temperature, feed concentration and feed rate had no significant influence on the Ru quantified in the process water. The steady-state concentrations were very similar and stable (bar the spikes) throughout the different testing phases. This followed the constant modelled Ru concentration between 350 °C and 500 °C. The mean concentrations at the different steady states were six to seven times higher than the modelled dissolution equilibrium values, independent of temperature or feed concentration. The Ru equilibrium concentration could be influenced by chelating anions formed by glycerol degradation products (when *X*_C_ ≪ 100%). However, this is not thought to occur since organometallic complexes are expected to decompose rapidly in SCW, as shown with metal-acetate complexes (*e.g.* Fe(Ac)_2_, Co(Ac)_2_, Ni(Ac)_2_) that decompose to the corresponding metal oxide and hydroxide in SCW.^49^ An iron citrate complex was also shown to completely decompose to Fe_3_O_4_.^66^ The comparison between the model and this work's data could indicate two distinct Ru loss mechanisms, the first one being leaching until solvent saturation, and the second being in the form of small Ru NP loss.

### Intentional pressure and feed rate fluctuations – influence on Ru loss

**Fig. 5:**
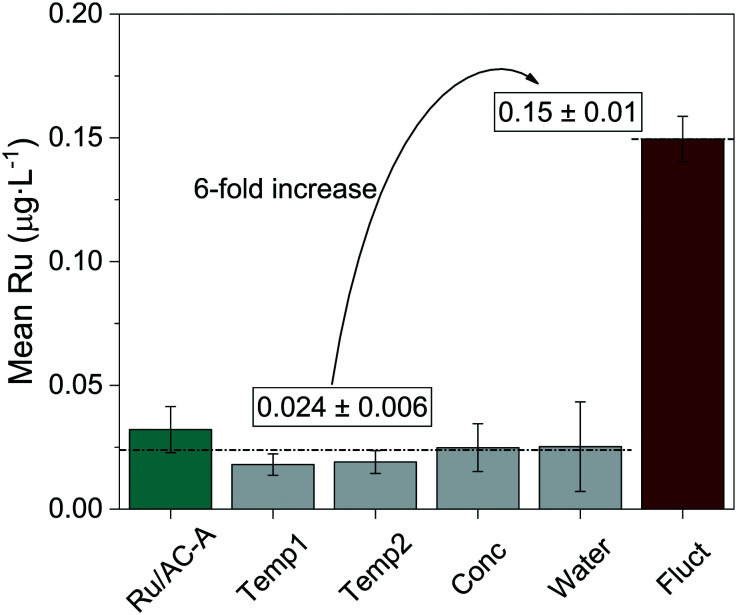
Comparison between the different SCWG experiments with the Ru/AC-A catalyst. The Ru concentrations were averaged for the different experiments at WHSV_g_Ru__ ≈ 500 g_Org_ g_Ru_^−1^ h^−1^. Ru/AC-A (dark cyan) represents the data from Fig. 2. Temp1, Temp2, Conc and Water (grey) were averaged from the parametric study (Fig. 3). Fluct (wine) shows the average Ru concentration after deliberately fluctuating the feed rate and pressure (10 wt% glycerol, 400 °C, TOS = 4 h). The horizontal dashed line represents the mean Ru concentration from the first five experiments (0.024 ± 0.006 μg L^−1^).

Decreases in both Al and Ru concentrations were systematically seen at the start of an experiment or after changes in process parameters. Concentration spikes were observed for Ru only and represented a sudden release of higher amounts of active phase. As Al concentration spikes were not observed, this could be explained by the physical stability of the materials, AC being less stable than α-Al_2_O_3_. These spikes could therefore be related to the loss of Ru particles through support disintegration, gasification or because of friction between the catalyst grains in the reactor. Impacts between catalyst grains can also occur if the catalyst bed is lifted up for short periods due to pressure or flow variations. The subsequent drop onto the wire mesh holding the catalyst bed would lead to considerable impacts on the catalyst grains and hence the loss of AC containing Ru NPs. To validate this hypothesis, an additional SCWG experiment was performed on Konti-I while applying deliberate changes in the feed rate (5–20 g min^−1^, 5 g min^−1^ step) and in the pressure (24–31 MPa, 0.1 MPa s^−1^ variation rate, Fig. S7†). Fig. 5 highlights the difference between experiments that were run “smoothly” (dark cyan and grey) and the deliberate fluctuation experiment (wine). The mean steady-state Ru concentration from the former SCWG experiments amounted to 0.024 ± 0.006 μg L^−1^ Ru, whereas a six-fold increase was observed when the process parameters were intentionally varied, reaching 0.15 ± 0.01 μg L^−1^ Ru. This is a strong indication that the hypothesis is valid and that repeated changes in the feed rate and pressure lead to a greater Ru loss. As impacts in the reactor can release Ru-containing carbon particles, the larger ones (>15 μm) will be trapped in the frit downstream of the reactor. To take this into account, the frit was sonicated in DI H_2_O before and after the intentional fluctuation experiment and the accumulated Ru was quantified. The initial amount of Ru found in the frit was 29 ng_Ru_, which accounted for the accumulation from all experiments performed before the fluctuation test (>250 h of SCWG). After the intentional fluctuation experiment, 298 ng_Ru_ were quantified in the frit. This ten-fold increase in Ru over a significantly shorter time emphasised the negative effect that brutal flow and pressure variations have on the mechanical stability of Ru/AC, and probably applies to other AC-supported catalysts too. Keeping the total TOS in mind for this Ru accumulation in the frit, “smooth” plant operation led to the loss of 29 ng_Ru_ in over 250 h of SCWG, whereas intentional fluctuations released 298 ng_Ru_ in only 3.8 h. Taking the total Ru loss into account (process water and frit accumulation) for the steady-state and fluctuation tests, 95% of the Ru loss can be ascribed to friction and collisions releasing small catalyst fragments. These results confirmed the initial hypothesis that impacts and friction between catalyst grains led to a mechanical loss of Ru.

### Impact of the continuous SCWG unit on Ru loss

**Fig. 6:**
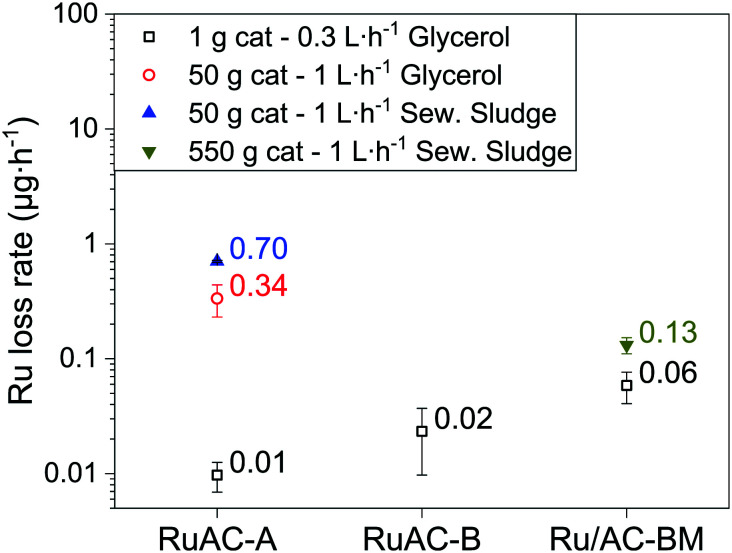
Ru loss overview for the commercial catalysts under different testing conditions. Empty symbols represent experiments with 10 wt% glycerol as feed. Full symbols represent tests with sewage sludge. The black squares relate to experiments performed on Konti-I, the red circle and blue triangle indicate results from the intermediate setup, where glycerol and real biomass were gasified. The dark yellow triangle represents results from Konti-C. The sampling strategy differed between the setups. When glycerol was processed (Konti-I and intermediate setups), the effluent was sampled for 3–5 min, once per hour. With sewage sludge (intermediate and Konti-C setups), the effluent was continuously collected and one sample represents an average concentration over a 3 h period.

Looking towards larger scale and industrialisation of the cHTG technology, it is very important to better understand the influence of the catalyst bed size (height and weight) on Ru loss and the catalyst's activity and stability when exposed to real biomass. For this purpose, the best-performing catalyst with the model feed (Ru/AC-A) was selected for tests on a 50 g catalyst bed reactor (intermediate setup). The characteristics of the different SCWG setups are shown in Table 1. On the intermediate setup, the experiment began by gasifying a 10 wt% glycerol solution until a steady state was maintained for 3 h. Afterwards, the feed was changed to water before pumping in sewage sludge. Fig. 6 shows a summary of the average steady-state Ru concentrations for the different experiments. It is important to keep in mind that the different setups used do not have the same reactor designs. The data points represented by black squares relate to the results from the smallest setup (Konti-I), where only aqueous glycerol solutions were gasified over a 1 g catalyst bed. The red and blue symbols come from the intermediate setup, where glycerol (red circle) and sewage sludge (blue triangle) were gasified over 50 g Ru/AC-A. The dark yellow triangle comes from PSI's 1 kg h^−1^ cHTG unit Konti-C^53^ (550 g Ru/AC-BM) operated with sewage sludge. Note that in both the intermediate and Konti-C setups, the catalyst beds were protected by a sulphur trap bed (upstream of the catalyst bed, analogous to α-Al_2_O_3_ in Konti-I) as well as a salt separator equipped with a brine extraction setup.^53^ The ZnO-based sulphur trap bed was shown to undergo attrition and leaching during 100 h of SCWG of microalgae, as Zn and Ca (binder matrix) were quantified in the Ru/AC catalyst bed downstream (7.7 and 0.1 wt% respectively).^55^ This can lead to fouling of the catalyst and eventually deactivation. The process water from the Konti-C experiment only showed 0.3 ppm Zn and 0.05 ppm Ca, indicating that most of the leached sulphur trap material would accumulate in the catalyst bed over long-term experiments. However, no deactivation was observed during the Konti-C test, as it was only run for 8 h with excess catalyst. The salt separator was used to remove the majority of the minerals by taking advantage of the low salt solubility in SCW, thus preventing rapid deactivation of the catalyst by fouling. The brine extraction setup was used for gasification experiments with sewage sludge. The salt separator efficiency was not 100% during both sewage sludge tests (intermediate and Konti-C setups), hence some inorganic compounds might have reached the catalyst bed. The lowest Ru concentration when gasifying glycerol on the Konti-I setup (Fig. 6, black squares) was measured for Ru/AC-A. Compared to Ru/AC-BM, the concentration was an order of magnitude smaller. As the catalysts were produced from different companies, it is supposed that the preparation techniques differ, highlighting the importance of the catalyst synthesis method. The stability of the support is very important too, as the brittleness of AC requires additional handling care (*e.g.* reactor filling procedure) to minimise impacts between catalyst grains and thus the potential loss of catalyst through friction and attrition. Typical crushing strengths of AC (≈10 MPa)^67^ are orders of magnitude below the ones of SCW-stable metal oxides (Al_2_O_3_, ZrO_2_, TiO_2_: 1000–5000 MPa).^68–70^

For the glycerol experiments with Ru/AC-A (Fig. 6), there was a 30-fold increase in Ru loss rate between the two setups, while there was 50 times more catalyst in the larger (intermediate) setup. This shows that different setup designs can affect the stability of the catalyst. When comparing the data from the intermediate setup, there was an additional two-fold increase in Ru loss rate between the model feed (red circle) and real biomass (blue triangle). Numerous factors could be the cause of this increase in Ru loss. One could think that changing from a model feed to real biomass may have an influence on the Ru dissolution equilibrium because of various inorganic and organic compounds reaching the catalyst. For instance, ammonia is expected to form during SCWG of manure (400 °C, 24 MPa) according to the thermodynamic model developed by Yakaboylu *et al.*^17^ Among the different process waters analysed there was no link between higher Ru concentration and higher ammonium content (determined by its counter-ion carbonate), hence ruling out the formation of stable complexes. Meanwhile, other molecules may still form Ru complexes of higher solubility. Corrosion is another phenomenon that can take place under SCW conditions, as some anions (*e.g.* nitrate, sulphide, sulphate) are powerful oxidisers and could lead to a higher Ru solubility, similarly to Ni.^71^ As sulphur was present in the sewage sludge feed (2.5 wt%, dry matter base) and quantified in the process water of Konti-C (0.34 ± 0.02 ppm), stable Ru sulphate complexes may have formed as reported by Waldner *et al.*^27^ Such complexes may have an increased solubility in SCW, which could explain the two-fold increase in Ru concentration between glycerol and sewage sludge gasification. XRD (Fig. S8†) spectra showed that no reflections were detected in Ru/AC-A after gasification of sewage sludge, confirming that the Ru NPs remained small (3–4 nm). Furthermore, no additional phases were formed after gasification of sewage sludge.

For Ru/AC-BM, the Ru loss rate was two times higher for sewage sludge gasification over 550 g catalyst than for glycerol gasification over 1 g catalyst. As for the intermediate setup, this difference could arise because of the formation of stable Ru complexes. Another aspect to keep in mind this time is that both reactors do not have the same design. Indeed, the catalyst bed height (H_cat_) was much larger in the Konti-C (and intermediate) setup than in Konti-I, leading to additional pressure on the lowest layer of the catalyst bed, amounting to 0.02, 0.07 and 0.08 bar for the Konti-I, intermediate and Konti-C setups, respectively (see eqn (S1)†). These pressures being orders of magnitude lower than the crushing strength of AC (≈10 MPa),^67^ the static forces alone can be ruled out, indicating that the friction between the catalyst grains along the reactor bed may be the main cause of additional Ru loss. The intrinsic pressure induced by the catalyst bed was similar for the intermediate and Konti-C setups, but the Ru loss rates were 5–6 times higher for both glycerol and sewage sludge gasification. As the reactors were different, one can think of the catalyst bed aspect ratio (H_cat_ d^−1^), relating the catalyst bed height over the reactor diameter as a reason for the increased Ru loss. The aspect ratios for the Konti-I, Konti-C and intermediate setups are equal to 6, 28 and 70, respectively. The larger height-to-diameter ratio of the intermediate setup could thus explain the higher Ru loss, since pressure or flow rate fluctuations would induce vertical movement in the catalyst bed and hence increased collisions between the catalyst grains. Larger H_cat_ d^−1^ would also lead to higher collision forces between the grains. It is important to note that the beds did not mix during experiments and could be recovered separately. In absolute concentration, the data point from the Konti-C setup was lower than both measured values (glycerol and sewage sludge) from the intermediate setup. Although the catalyst used in Konti-C was not fresh and had already experienced more than 120 h of SCWG, it should not affect the data interpretation as the Ru concentration remained stable for more than 50 h (Fig. 2). No correlation was observed between the Ru loss and the mass of catalyst loaded (Fig. S9†), residence time (Fig. S10, right†) or superficial velocity (Fig. S10, left†). The fact that no correlation was found between the total amount of catalyst and the Ru concentration in the process water indicated that Ru leaching was the dominating Ru loss mechanism at steady state (limited only by Ru solubility), which is in line with the time-resolved ICP-MS data (Fig. S2–S4†).

**Fig. 7:**
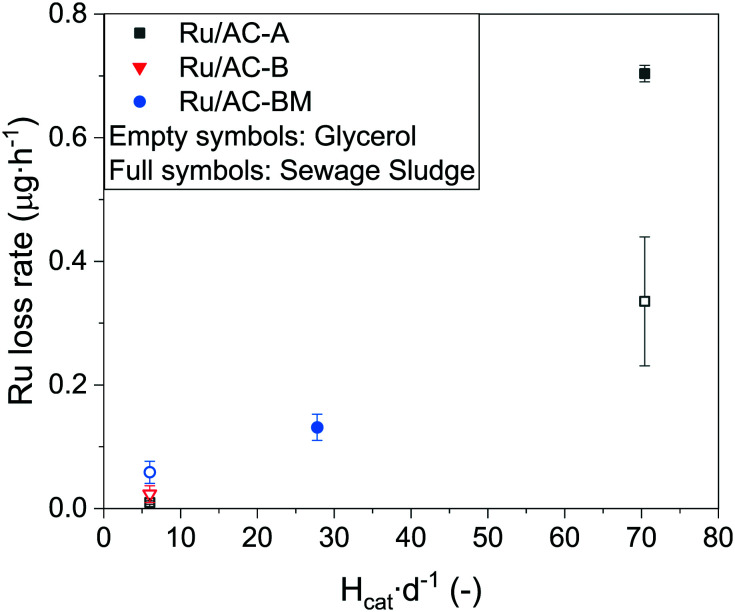
Ru loss rate in the effluent stream as a function of catalyst bed aspect ratio. Aspect ratios (H_cat_ d^−1^) of 6, 28 and 70 correspond to the Konti-I, Konti-C and intermediate setups, respectively. Empty symbols represent experiments with 10 wt% glycerol, full symbols with sewage sludge. Process conditions (from Table 1): Konti-I (glycerol) WHSV_g_Ru__ = 620 g_Org_ g_Ru_^−1^ h^−1^, intermediate (glycerol and sewage sludge) WHSV_g_Ru__ = 16 and 5 g_Org_ g_Ru_^−1^ h^−1^, Konti-C (sludge) WHSV_g_Ru__ = 0.4 g_Org_ g_Ru_^−1^ h^−1^.

Fig. 7 shows a correlation between the Ru loss rate and H_cat_ d^−1^, indicating that collisions, friction and attrition induced by pressure and flow rate fluctuations may participate as Ru loss mechanisms. The difference between glycerol and sewage sludge gasification in the intermediate setup may arise from the different chemistry, as impurities (*e.g.* sulphur) in sewage sludge can form stable, more soluble Ru complexes. For example, Osada *et al.*^72^ reported that sulphur addition during SCWG of lignin over Ru catalysts led to the formation of Ru–S complexes (*e.g.* sulphide, sulphite, sulphate). Other studies also reported the presence of sulphate^27,33,73^ and sulphide^33^ after SCWG with Ru-based catalysts. The steady-state Ru concentrations for all Ru/AC experiments were in the same range (0.01–0.03 μg L^−1^). As stated previously, no correlation was found between the quantity of Ru in the reactor and the Ru concentration in the process water, independent of the conditions and catalyst loading (Fig. S9†). This indicates that the Ru loss from the catalyst at steady state was close to the thermodynamic equilibrium, *i.e.* absence of concentration gradient (driving force for dissolution). The absence of correlation between the feed rate and absolute Ru concentration (Fig. S11†) indicated that NP detachment from the support did not have a significant impact on the global Ru loss, as one would expect a dilution of Ru NPs with an increasing flow. Although higher flow rates could lead to more Ru NPs detaching from the support, the Ru concentration trends for the three catalysts are in complete opposition (Fig. S11†), supporting the initial hypothesis. The results support a dominating occurrence of Ru leaching at levels higher than predicted by models, together with a lower probability of Ru loss through NP detachment. However, Fig. S12† indicates that the modelled leaching would only account for 2–6% of the total Ru loss during 10 wt% glycerol gasification of the three commercial 5 wt% catalysts. This would mean that the main loss mechanisms would be through small Ru NPs instead of leaching, which is not thought to be the case. Nevertheless, the measured steady-state Ru loss by leaching is extremely low; meaning that mechanical Ru loss by friction/abrasion induced by variations in process parameters was the main cause of Ru loss from the catalyst bed.

### Ru loss from 2 wt% Ru catalysts

The first hypothesis was that the spikes in Ru concentration originated from the loss of support through fragmentation due to the brittleness of AC, as well as from the gasification of activated carbon by the Ru NPs. Despite the kinetics being very slow, support gasification is thermodynamically favoured under SCW conditions.^43,74,75^ This hypothesis was already partly answered in the previous section by showing the increased Ru loss during brutal SCWG operation. However, it is still unclear whether Ru/AC is stable enough under steady state SCWG conditions compared to its metal oxide analogues, and whether the increased physico-chemical stability of some refractory metal oxides could lead to lower Ru loss. To confront this, Ru catalysts were synthesised on three SCW-stable metal oxide supports.^23^ Because of the low pore volumes and surface areas of the metal oxides, the targeted metal loading was decreased to 2 wt% Ru. The main catalyst properties as well as the WHSV_g_Ru__ at which the tests were run are summarised in Table 4.

**Table 4:** Characteristics of the synthesised 2 wt% Ru catalysts with experiment WHSV_g_Ru__

Catalyst	*x*_Ru_ a (%)	*D* b (%)	*d*_p_ c (nm)	SSA_Tot_ (m^2^ g^−1^)	SSA_Ext_ (m^2^ g^−1^)	WHSV_g_Ru__ (g_Org_ g_Ru_^−1^ h^−1^)
2% Ru/AC	2.30	72	1.3	1354	136	1370
2% Ru/α-Al_2_O_3_	1.74	3	52	6.6	5.7	1511
2% Ru/r-TiO_2_	1.74	1	104	4.5	3.7	1724
2% Ru/m-ZrO_2_	1.80	40	2.6	26.9	22.0	1604

a Loading determined by Ru mass balance.

b Ru dispersion measured by CO pulse titration.

c Calculated from *D* according to ref. 53.

**Fig. 8:**
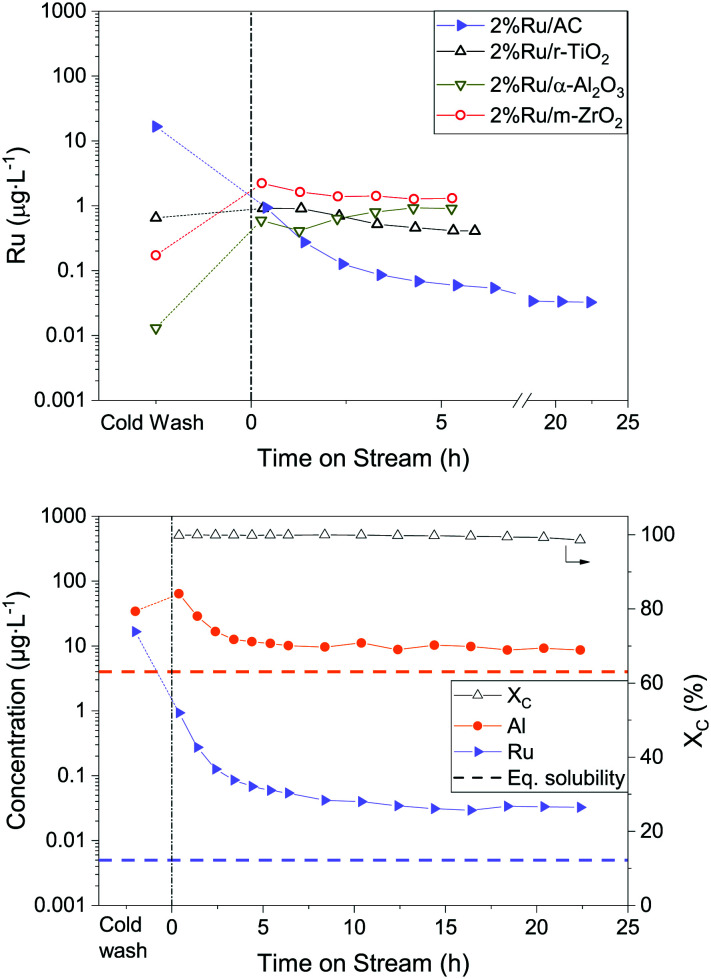
Ru concentration in the effluent stream as a function of time on stream for the 2% Ru catalysts. Left: Ru/AC (blue), Ru/r-TiO_2_ (black), Ru/m-ZrO_2_ (red), Ru/α-Al_2_O_3_ (dark yellow). Right: Carbon conversion (empty triangle), Al (orange circle) and Ru (blue triangle) concentrations from the 2% Ru/AC SCWG experiment. 10 wt% glycerol was fed at TOS = 0 h. The blue triangles in both graphs represent the same data.

Although the WHSV_g_Ru__ was too high to sustain long thermodynamic gas equilibria, the carbon conversion remained above 99.9% for almost 20 h with the AC-supported Ru catalyst (Fig. S13†). The methane yield remained constant at 28 g_CH_4__ g_gly_^−1^, which is the thermodynamic limit for this system.^23^ Regarding the metal oxides, none of them achieved total conversion – the decrease was very rapid over the first 2–3 hours, as already reported in other works.^23,31^ Low surface area supports such as titania and alumina led to poor Ru dispersions (Ru particle diameters of 104 and 52 nm, respectively), explaining their bad performance. However, the zirconia-based catalyst showed a mean Ru NP diameter of 2.6 nm (comparable to 2% Ru/AC), but remained only marginally more active than the other metal oxides and much less than the AC-supported catalyst after 5 h of glycerol gasification. This could mean that the Ru particle size (dispersion) alone was not responsible for the good gasification activity of these Ru-based catalysts. The results of the ICP-MS analyses of the process water are reported in Fig. 8. As shown in the left-hand side graph, the Ru concentration for 2% Ru/AC was the only one that exhibited a sharp decrease starting from the cold wash. Conversely, the metal oxide-supported catalysts showed low Ru concentrations during the cold wash, and then increased when the setup was under SCWG conditions (TOS = 0 h). The steady-state Ru concentrations measured in the process water were more than one order of magnitude higher for the metal oxides (*ca.* 1 μg L^−1^) than for the AC-based catalyst (0.04 μg L^−1^). Converted into normalised Ru loss rates, they amounted to 10.3 ± 3.7, 24.3 ± 5.6 and 10.2 ± 2.9 μg g_Ru_^−1^ h^−1^ for 2% Ru/TiO_2_, 2% Ru/Al_2_O_3_ and 2% Ru/ZrO_2_ at steady state, respectively. This is considerably higher than the 0.5 ± 0.1 μg g_Ru_^−1^ h^−1^ of 2% Ru/AC. Li *et al.*^59^ reported that the difference in metal loss rates between Ni/α-Al_2_O_3_ and Ni/CNT (carbon nanotubes) (10^−3^*vs.* 0.5 × 10^−3^ g_Ni,lost_ g_Ni,bed_^−1^ h^−1^) during SCWG of glycerol was due to the fact that smaller metal NPs have proportionally more atoms in contact with the support and hence leach less. This statement would hold true for 2% Ru/TiO_2_ (Fig. S14†) and 2% Ru/Al_2_O_3_ (Fig. S15†), but not for 2% Ru/ZrO_2_ (Fig. S16†), which exhibited small Ru NPs but high Ru loss. The cold wash Ru concentration (16.6 μg L^−1^) of 2% Ru/AC was significantly higher than the average concentration at steady state (0.04 μg L^−1^) and therefore led to a higher overall Ru loss compared to the metal oxide catalysts. Over the whole experiment duration, the cold wash contributed to 94% of the Ru loss for 2% Ru/AC (0.6% for Ru/α-Al_2_O_3_, 13% for Ru/TiO_2_ and 4% for Ru/ZrO_2_). This shows that the least stable carbon domains and weakly attached Ru NPs were washed out of the reactor in the initial phase. However, the Ru loss rapidly stabilised to very low concentrations for 2% Ru/AC, which was not the case for the metal oxide catalysts. Although the initial Ru loss was high for 2% Ru/AC, it did not contribute to the deactivation of the catalyst as shown in Fig. 8.

One might argue that the very high surface area of activated carbon can act as a filter or a trap and can readsorb the leached metals or the detached Ru NPs. This theory is invalidated by the fact that Al leached from the α-Al_2_O_3_ filling material into the process water at similar levels (close to modelled levels), be it in the presence downstream of the low surface area 2% Ru/TiO_2_ (Fig. S13†) or the high surface area 2% Ru/AC catalyst. One possible explanation for the greater Ru loss from metal oxide catalysts could be the weaker metal–support interaction. In stable conditions, the brittle AC remains an optimal catalyst support for continuous SCWG: not only was the steady-state Ru loss 20 times lower with AC compared to the tested metal oxide-based catalysts, but it also showed the highest activity.

## Conclusions

In this work, we were able to measure very low Ru concentrations in SCWG process water by time-resolved ICP-MS. From these data, we conclude that the active phase loss from Ru/AC catalysts during SCWG is governed by several mechanisms in parallel:

1. Constant Ru dissolution until solubility equilibrium is reached (steady state);

2. Minor NP uncoupling from the support (steady state);

3. Support disintegration leading to the loss of larger amounts of Ru caused by abrupt feed rate or pressure variations.

We showed that steady-state Ru leaching from Ru/AC catalysts is the lowest (compared to Ru/metal oxides) and is very close to the reported thermodynamic limit. There is hence very little room for improvement in their leaching resistance. This serves as additional evidence that Ru/AC is the best catalytic system for SCWG. Mechanical damage to the Ru/AC catalyst grains led to the highest Ru losses (up to 100-fold increase), emphasising that this loss mechanism must absolutely be avoided.

Looking ahead towards industrialisation of this technology, smooth plant operation is vital to prevent catalyst damage and consequently significant Ru loss through friction or attrition. This learning is pivotal to guarantee long catalyst lifetimes in commercial cHTG plants and prevent process water contamination by ruthenium.

## Conflicts of interest

There are no conflicts to declare.

## Supplementary Material

CY-011-D1CY00379H-s001
