# Supplementary material for: Investigating active phase loss from supported ruthenium catalysts during supercritical water gasification
Source: Catal Sci Technol. 2021 Oct 14;11(22):7431–44. doi: 10.1039/d1cy00379h (PMC8591986; doi:10.1039/d1cy00379h)
Supplement: CY-011-D1CY00379H-s001 [file CY-011-D1CY00379H-s001.pdf]

Electronic Supplementary Material (ESI) for Catalysis Science & Technology.
This journal is © The Royal Society of Chemistry 2021

Investigating Active Phase Loss from Supported Ruthenium Catalysts during Supercritical Water Gasification

C. Hunston,[a,b] D. Baudouin,*[a] M. Tarik,[a] O. Kröcher,[a,b] and F. Vogel[a,c]

[a]Bioenergy and Catalysis Laboratory, Paul Scherrer Institut (PSI), 5232 Villigen PSI, Switzerland.
[b]Institute of Chemical Sciences and Engineering (ISIC), École Polytechnique Fédérale de Lausanne (EPFL), 1015 Lausanne, Switzerland.
[c]Institute for Biomass and Resource Efficiency, Fachhochschule Nordwestschweiz (FHNW), 5210 Windisch, Switzerland.
*Corresponding author: david.baudouin@psi.ch

Electronic Supplementary Information

Table S1 ICP-MS instrument parameters and calibration parameters for ^{99}Ru and ^{101}Ru

ICP-MS parameters	Value
Power (W)	1350
Flow of carrier gas (Ar) (L·min^{-1})	0.94
Sampling depth (mm)	10
He flow (collision gas) (mL·min^{-1})	0
Integration time/isotope (s)	0.2
Acquisition time/sample (s)	70
**Calibration parameters**	
For ^{99}Ru	
Sensitivity (cps·ppt^{-1})	13.18
R^2	1.0000
LOD (pg·mL^{-1})	1.14
BEC (background equivalent concentration) (ppt)	3.60
For ^{101}Ru	
Sensitivity (cps·ppt^{-1})	19.17
R^2	1.0000
LOD (ppt)	1.08
BEC (background equivalent concentration) (ppt)	1.83

Table S2 Modelled thermodynamic equilibrium solubilities of different materials in SCW

	Compound	Log(Solubility)[a] (mol·kg$_{H_2O}^{-1}$)	Solubility (g·g$_{H_2O}^{-1}$)
Calculated	α-Al_2O_3	-7.13	$7.56\cdot10^{-9}$
	Al	-7.13	$4.00\cdot10^{-9}$
	RuO_2	-14.47	$4.51\cdot10^{-16}$
	TiO_2	-12.65	$1.79\cdot10^{-14}$
	ZrO_2	-9.99	$1.26\cdot10^{-11}$
Extrapolated[b]	Ru	-10.5	$3.20\cdot10^{-12}$
	RuO_2	-15	$1.33\cdot10^{-16}$
	TiO_2	-13	$7.99\cdot10^{-15}$
	ZrO_2	-10	$1.23\cdot10^{-11}$

[a] Data from Jocz et al.[1]
[b] Extrapolated from the graphs in the ESI of Jocz et al.[1]

The pressure on the lowest layer of the catalyst bed was calculated according to Equation S1:

$$p\,(bar) = p_{bed} + p_{H_2O} = \frac{m_{cat+fill} \cdot g}{A_{CS}} + \rho_{p,T} \cdot g \cdot H_{reactor} \tag{S1}$$

by taking into account the weight of the catalyst and filling material ($m_{cat+fill}$), the reactor cross-section area A_{CS}, as well as the weight contribution from SCW (400 °C, 29 MPa) of density $\rho_{29,400}$ = 0.31 g·mL^{-1} and the reactor height $H_{reactor}$ (from the top down to the end of the catalyst bed).

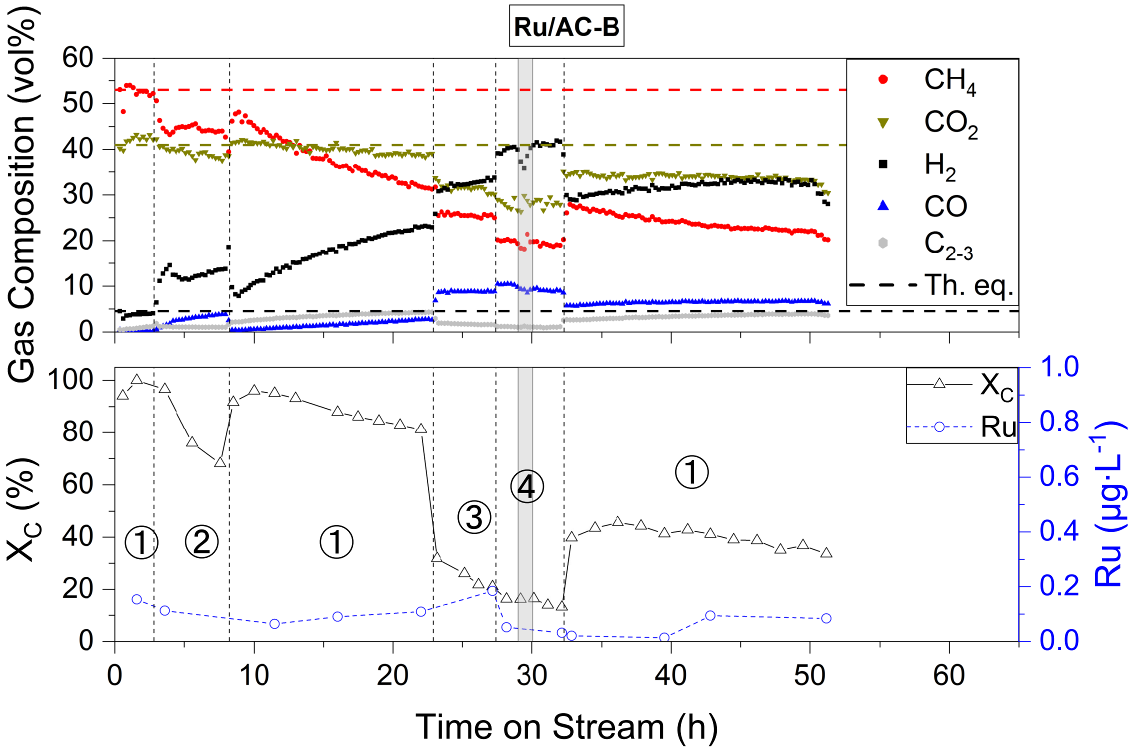

Figure S1 Catalyst testing overview for Ru/AC-B. The produced gas (top part of the graphs) and both the carbon conversion and Ru concentration (bottom part of the graphs) are shown as a function of the time on stream (glycerol fed from TOS = 0 h onwards). Horizontal dashed lines indicate the thermodynamic equilibrium gas composition. Conditions: T = 400 °C, p = 29 MPa, $WHSV_{gRu}$ ≈ 600, 1200, 1800, 2400 $g_{Org} \cdot g_{Ru}^{-1} \cdot h^{-1}$ for sections 1 – 4, respectively. A pump issue led to a pressure and temperature loss, represented by the grey area: p_{min} = 12 MPa, T_{min} = 340°C.

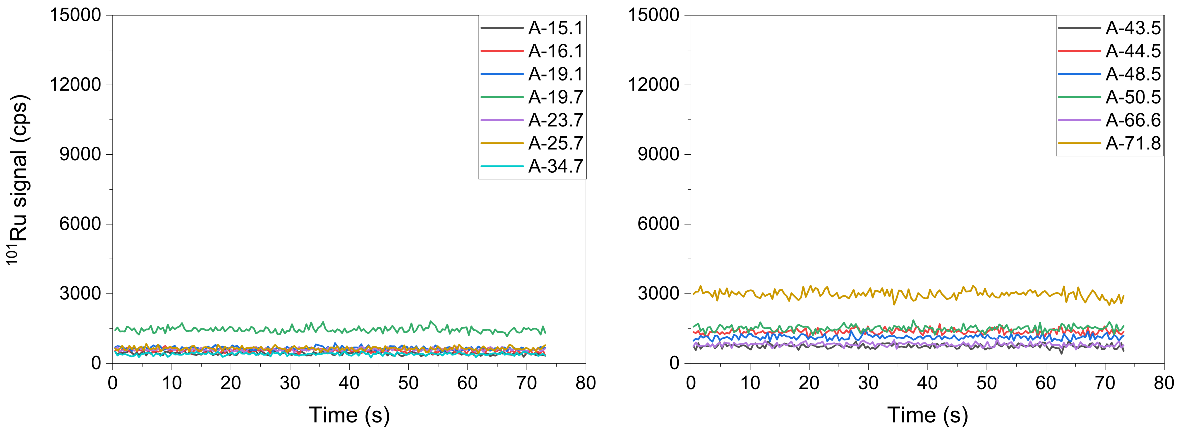

Figure S2 ICP-MS time-resolved analysis for selected Ru/AC-A SCWG samples. A-xx.x refers to the sample TOS (h).

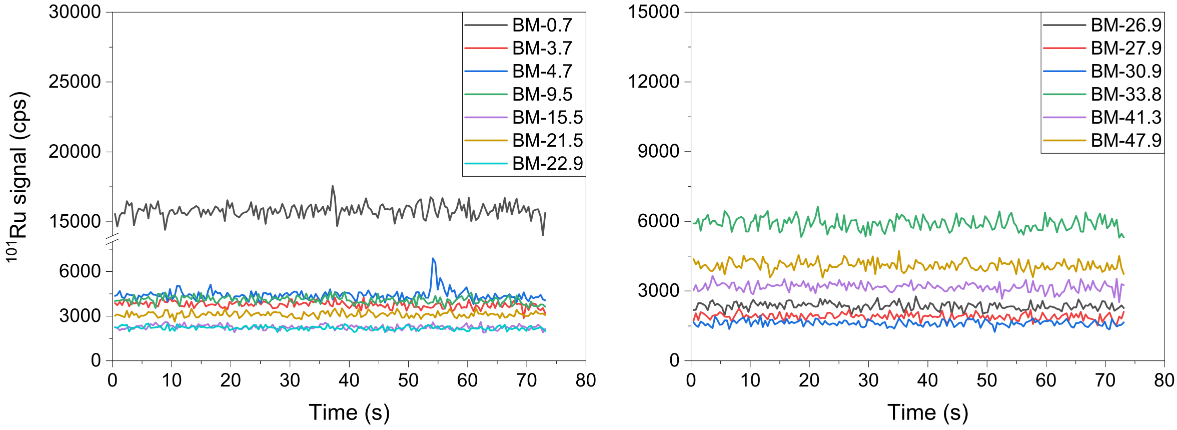

Figure S3 ICP-MS time-resolved analysis for selected Ru/AC-BM SCWG samples. BM-xx.x refers to the sample TOS (h).

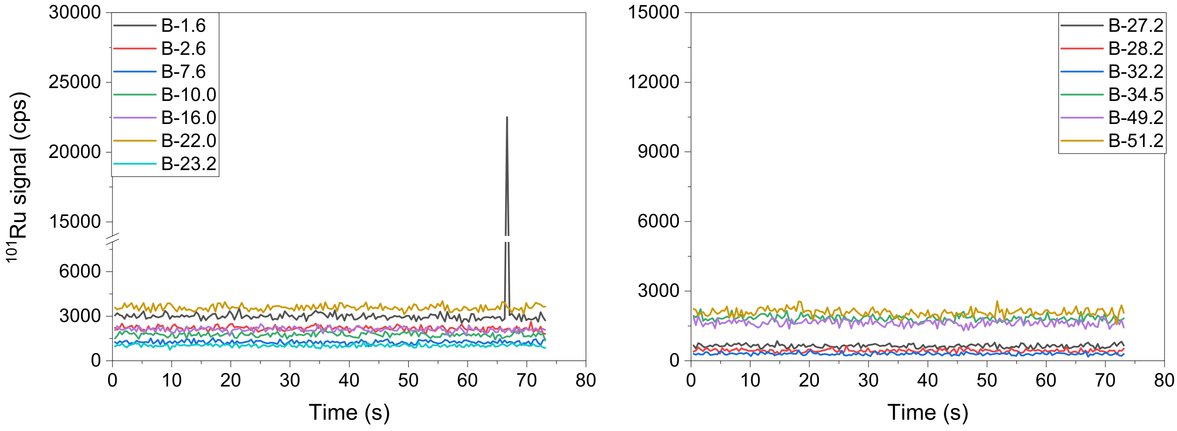

Figure S4 ICP-MS time-resolved analysis for selected Ru/AC-B SCWG samples. B-xx.x refers to the sample TOS (h).

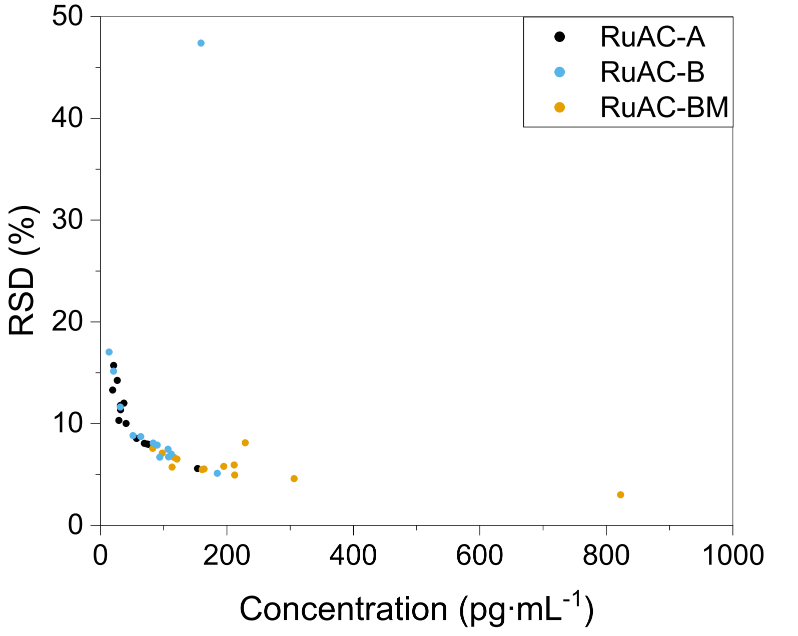

Figure S5 RSD of the time-resolved ICP-MS signals as a function of concentration for the three commercial Ru/AC catalysts. A main outlier is seen with Ru/AC-B-1.6 (Figure S4) and smaller outlier with Ru/AC-BM-4.7 (Figure S3).

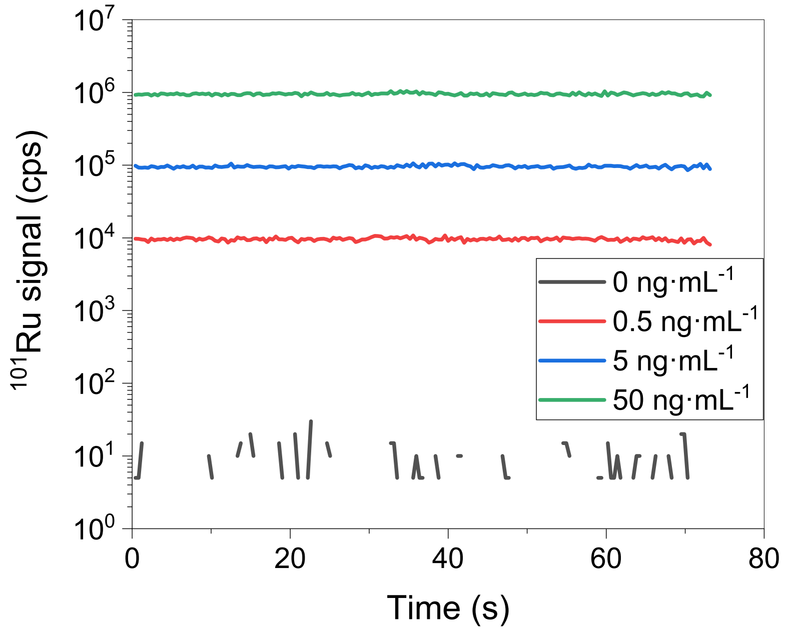

Figure S6 ICP-MS time-resolved signal of the ionic Ru standards used for the calibration. RSD = 4.7, 3.9 and 3.5 % for the 0.5, 5 and 50 ng·mL^{-1} standard concentrations, respectively. The line of the blank sample (0 ng·mL^{-1}) is discontinued because the measured signal is often 0.

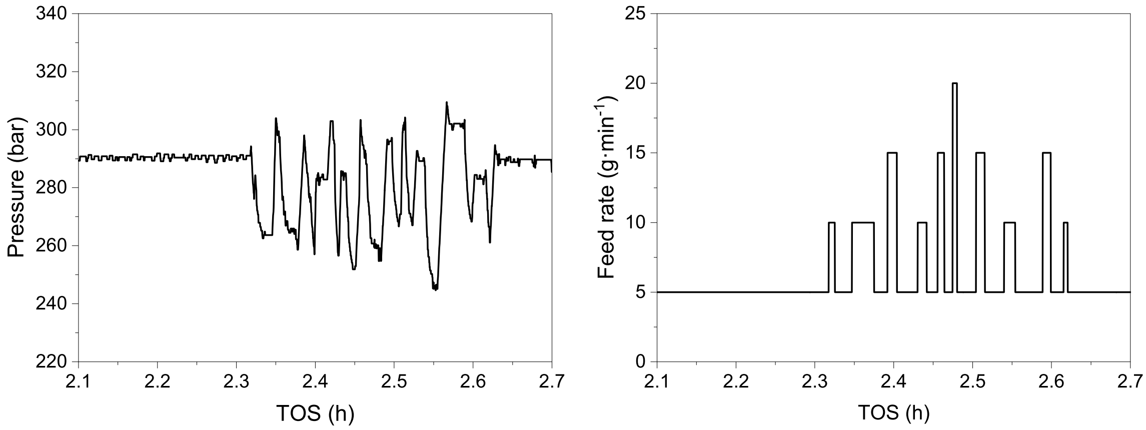

Figure S7 Pressure (left) and mass flow rate (right) variations during the intentional fluctuations test.

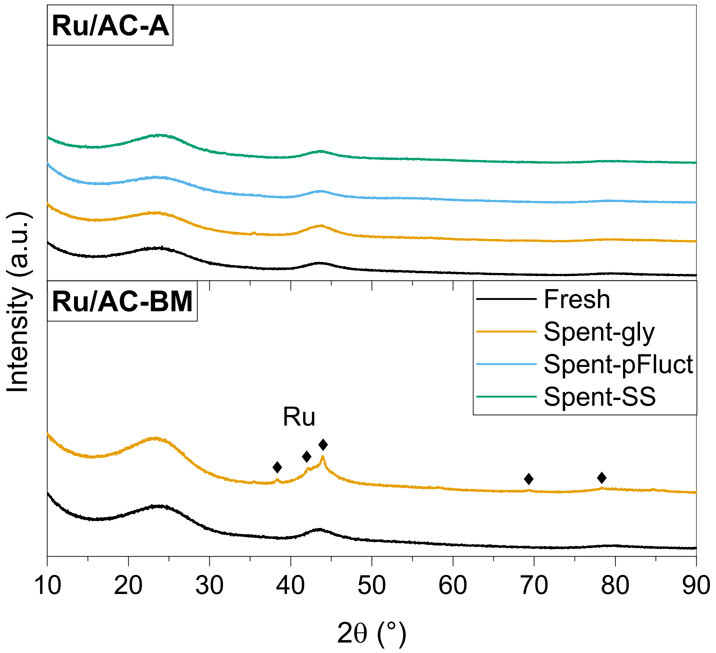

Figure S8 XRD spectra of fresh and spent samples of Ru/AC-A (top) and Ru/AC-BM (bottom). Spent-gly and Spent-pFluct refer to catalysts treated with glycerol on *Konti-I* in normal operation and during the deliberate fluctuations test, respectively. Spent-SS refers to the experiment on the *intermediate* setup with sewage sludge.

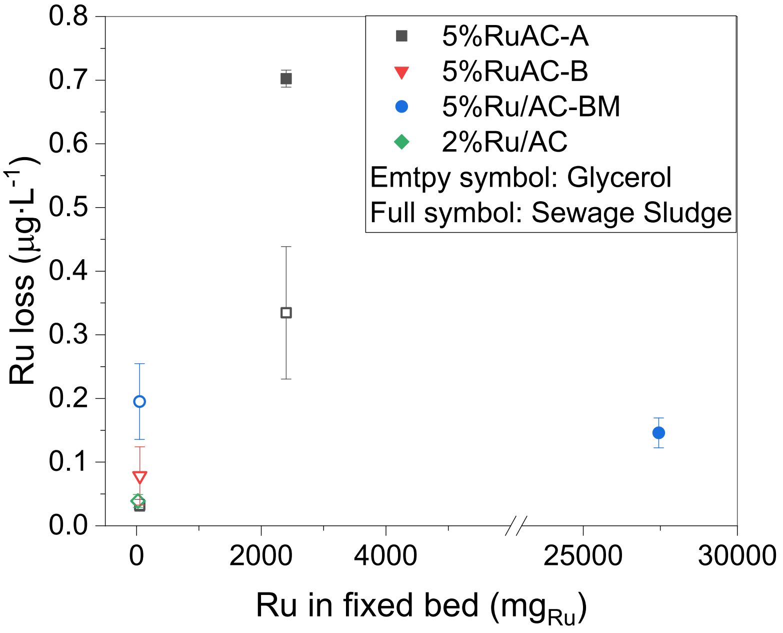

Figure S9 Ru concentration as a function of the total Ru loaded in the catalytic reactor for the three SCWG setups.

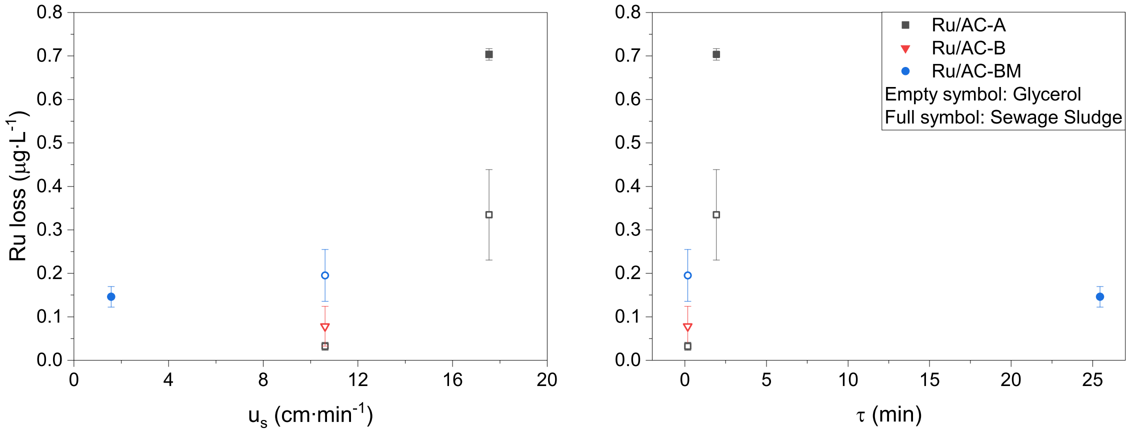

Figure S10 Ru concentration as a function of superficial velocity u_s (left) and residence time τ (right) in the catalyst bed for the three SCWG setups.

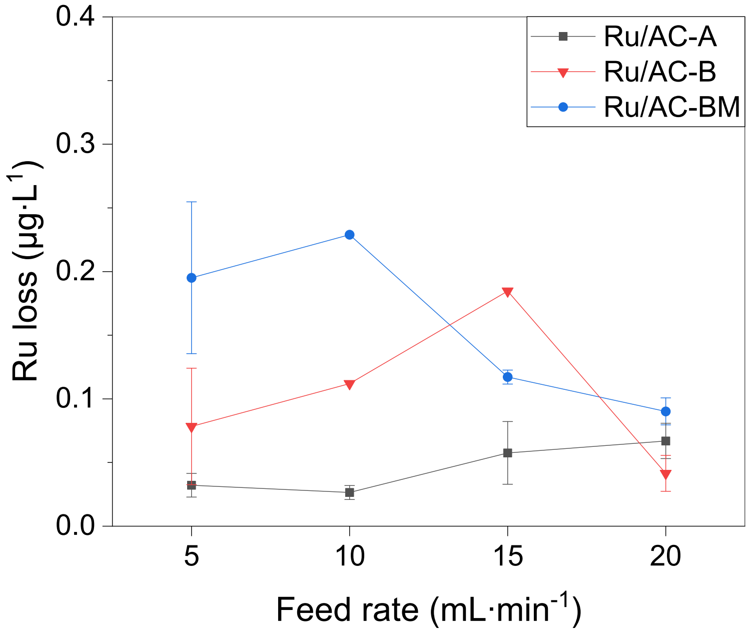

Figure S11 Ru concentration in the process waters from the three commercial 5 wt% Ru/AC catalysts as a function of feed rate for SCWG of glycerol on *Konti-I* (400 °C, 29 MPa).

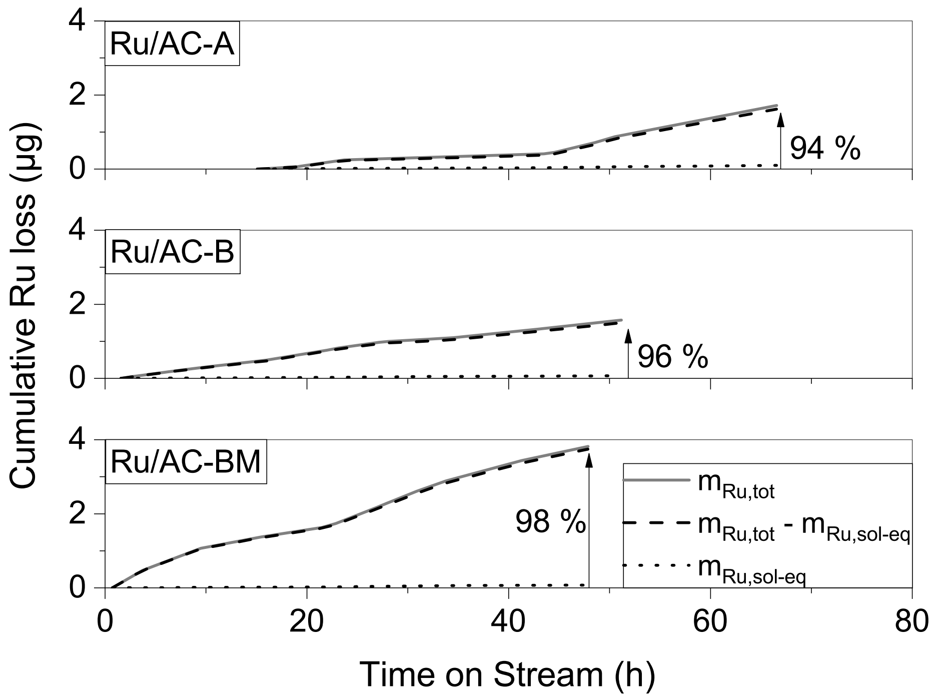

Figure S12 Cumulative Ru loss for the three commercial 5%Ru/AC catalysts. Total Ru loss ($m_{Ru,tot}$, grey line), Ru loss without the contribution of the equilibrium solubility *i.e.* leaching ($m_{Ru,tot} - m_{Ru,sol,eq}$, dashed line) and equilibrium Ru solubility[1] ($m_{Ru,sol,eq}$, dotted line). The number (%) refers to the percent of Ru lost without the contribution of the equilibrium solubility.

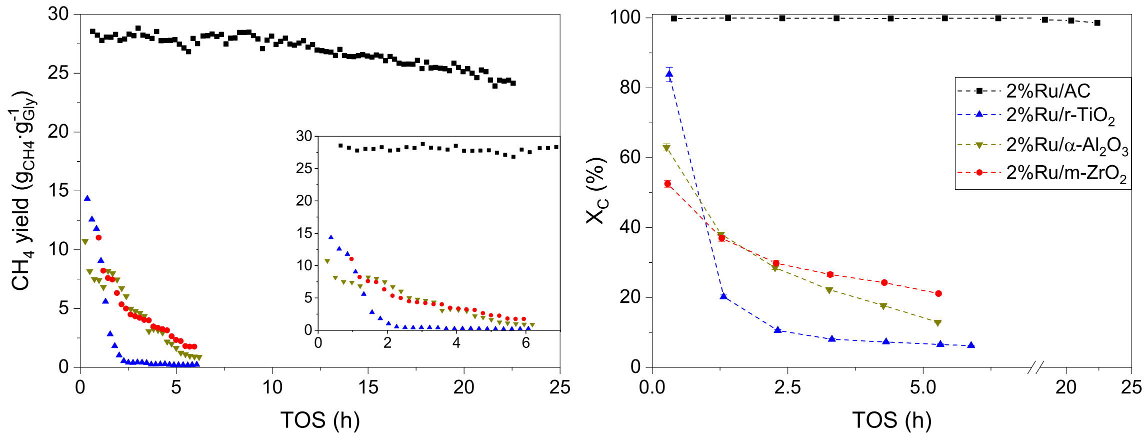

Figure S13 Catalytic performance of the synthesised 2%Ru catalysts. Left: methane yield as a function of time on stream. Right: carbon conversion as a function of time on stream (black: Ru/AC, blue: Ru/r-TiO_2, dark yellow: Ru/α-Al_2O_3, red: Ru/m-ZrO_2). Results from Zöhrer et al.[2] (same synthesis procedure, except the higher calcination temperature (560 °C) used by Zöhrer et al.) showed total conversion for a 2%Ru/m-ZrO_2 with glycerol at a $WHSV_{gRu}$ of only 30 $g_{Org} \cdot g_{Ru}^{-1} \cdot h^{-1}$. The results shown here were similar to the findings of Peng et al.[3] The main difference is the model substance used as feed, they used isopropanol instead of glycerol. This is reflected in the difference in deactivation rates, which were much higher with glycerol than with isopropanol.

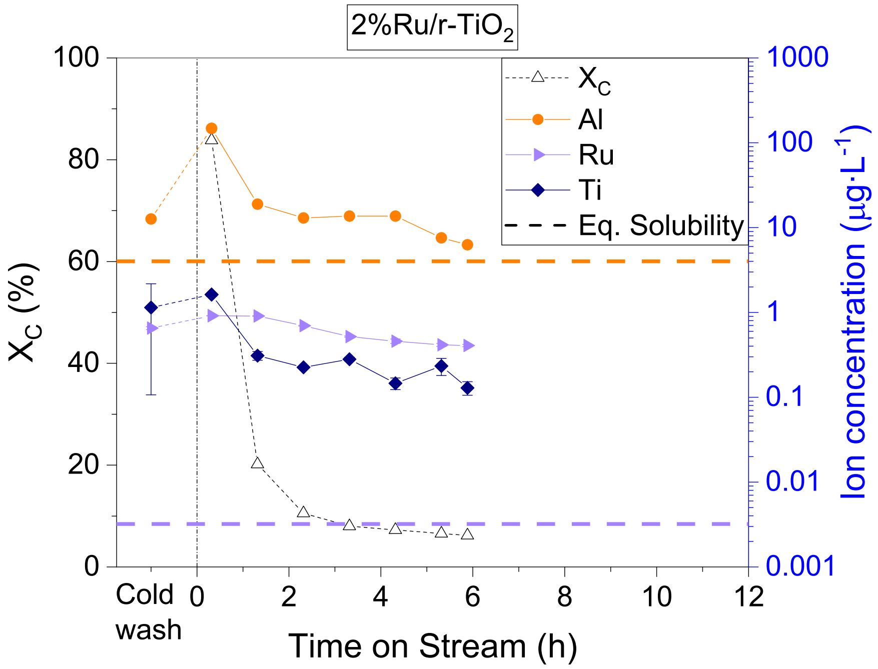

Figure S14 Conversion (black triangles), Al (orange circles), Ru (blue triangles) and Ti (navy blue diamonds) concentration in the process water as a function of time on stream for 2%Ru/TiO_2. Glycerol fed at TOS = 0 h. Horizontal dashed lines represent the metal dissolution equilibrium (Table S2) for the given species (not shown for Ti because its concentration is too low ($2{\cdot}10^{-5}$ $\mu g{\cdot}L^{-1}$).

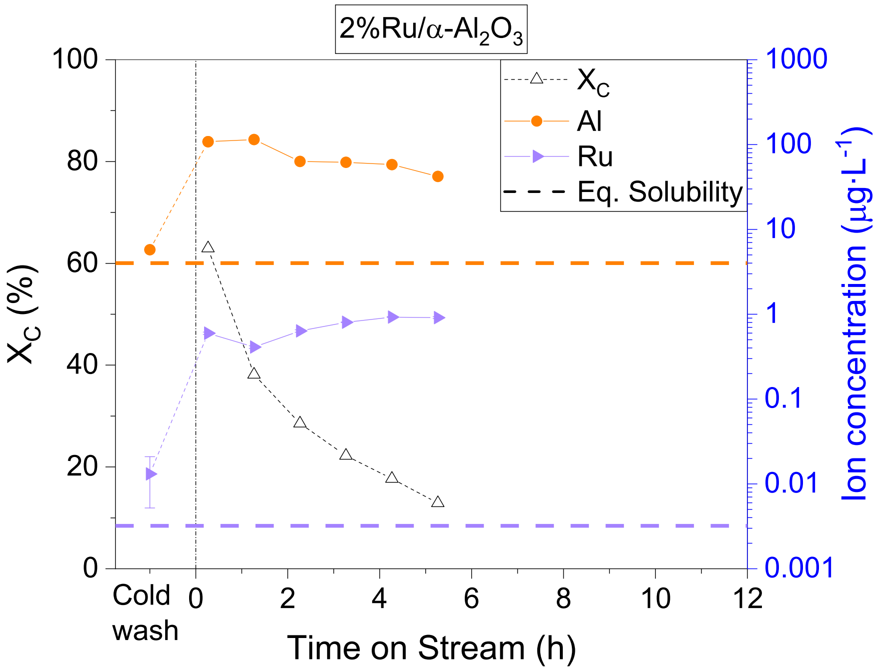

Figure S15 Conversion (black triangles), Al (orange circles) and Ru (blue triangles) concentration in the process water as a function of time on stream for 2%Ru/α-Al_2O_3. Glycerol fed at TOS = 0 h. Horizontal dashed lines represent the metal dissolution equilibrium (Table S2) for the given species.

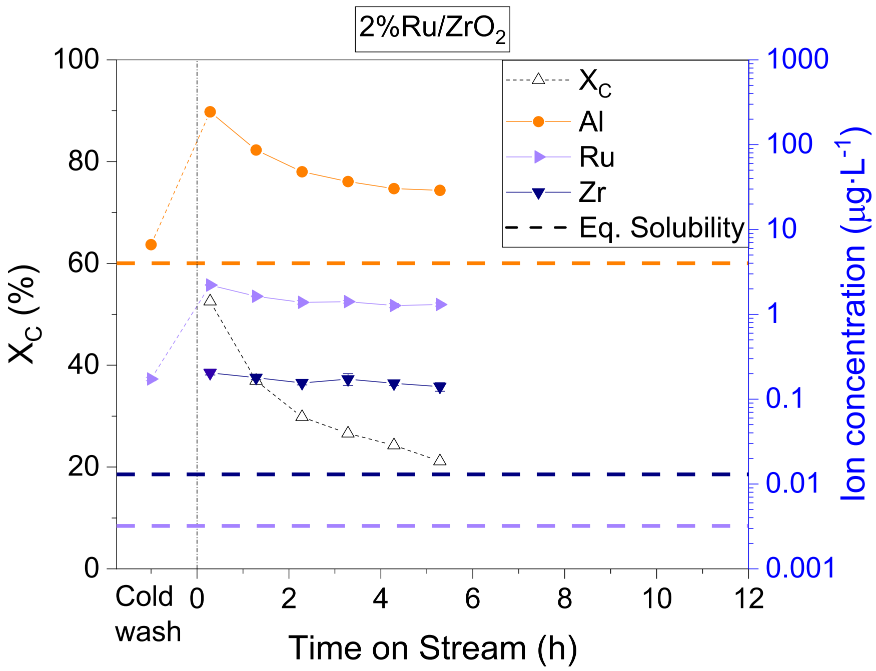

Figure S16 Conversion (black triangles), Al (orange circles), Ru (blue triangles, facing right) and Zr (navy blue triangles, facing down) concentration in the process water as a function of time on stream for 2%Ru/ZrO_2. Glycerol fed at TOS = 0 h. Horizontal dashed lines represent the metal dissolution equilibrium (Table S2) for the given species.

References

[1] J. N. Jocz, P. E. Savage and L. T. Thompson, *Ind. Eng. Chem. Res.*, 2018, **57**, 8655–8663.

[2] H. Zöhrer, F. Mayr and F. Vogel, *Energy Fuels*, 2013, **27**, 4739–4747.

[3] G. Peng, C. Ludwig and F. Vogel, *ChemCatChem*, 2016, **8**, 139–141.
